# Mammalian E-type Cyclins Control Chromosome Pairing, Telomere Stability and CDK2 Localization in Male Meiosis

**DOI:** 10.1371/journal.pgen.1004165

**Published:** 2014-02-27

**Authors:** Laetitia Martinerie, Marcia Manterola, Sanny S. W. Chung, Sunil K. Panigrahi, Melissa Weisbach, Ana Vasileva, Yan Geng, Peter Sicinski, Debra J. Wolgemuth

**Affiliations:** 1 Departments of Genetics & Development, Columbia University Medical Center, New York, New York, United States of America; 2 Center for Radiological Research, Columbia University Medical Center, New York, New York, United States of America; 3 Department of Genetics, Harvard Medical School and Department of Cancer Biology, Dana-Farber Cancer Institute, Boston, Massachusetts, United States of America; 4 Obstetrics & Gynecology, Columbia University Medical Center, New York, New York, United States of America; 5 Institute of Human Nutrition, Columbia University Medical Center, New York, New York, United States of America; Cornell University, United States of America

## Abstract

Loss of function of cyclin E1 or E2, important regulators of the mitotic cell cycle, yields viable mice, but E2-deficient males display reduced fertility. To elucidate the role of E-type cyclins during spermatogenesis, we characterized their expression patterns and produced additional deletions of *Ccne1* and *Ccne2* alleles in the germline, revealing unexpected meiotic functions. While *Ccne2* mRNA and protein are abundantly expressed in spermatocytes, *Ccne1* mRNA is present but its protein is detected only at low levels. However, abundant levels of cyclin E1 protein are detected in spermatocytes deficient in cyclin E2 protein. Additional depletion of E-type cyclins in the germline resulted in increasingly enhanced spermatogenic abnormalities and corresponding decreased fertility and loss of germ cells by apoptosis. Profound meiotic defects were observed in spermatocytes, including abnormal pairing and synapsis of homologous chromosomes, heterologous chromosome associations, unrepaired double-strand DNA breaks, disruptions in telomeric structure and defects in cyclin-dependent-kinase 2 localization. These results highlight a new role for E-type cyclins as important regulators of male meiosis.

## Author Summary

Understanding the control of meiosis is fundamental to deciphering the origin of male infertility. Although the mechanisms controlling meiosis are poorly understood, key regulators of mitosis, such as cyclins, appear to be critical. In this regard, male mice deficient for cyclin E2 exhibit subfertility and defects in spermatogenesis; however, neither the stages of germ cell differentiation affected nor the responsible mechanisms are known. We investigated how E-type cyclins control male meiosis by examining their expression in spermatogenesis and the consequences that multiple deletions of *Ccne1* and *Ccne2* alleles produce. Loss of *Ccne2* expression increases cyclin E1 levels as a compensatory effect, but there are still meiotic defects and subfertility. Further, loss of one *Ccne1* allele in the absence of cyclin E2 results in infertility as does loss of the remaining *Ccne1* allele, but with even more severe meiotic abnormalities. We further found that cyclin E1 is involved in sex chromosome synapsis while E2 is involved with homologous pairing and chromosome and telomere integrity. These processes and structures were severely disrupted in absence of both cyclin E1 and E2, uncovering new roles for the E-type cyclins in regulating male meiosis.

## Introduction

Cyclins are key cell cycle regulatory subunits that bind, activate, and provide substrate specificity for the cyclin-dependent kinases (CDKs). Although the role of cyclins in the somatic mitotic cycle has been extensively studied, their function in the meiotic cycle is poorly understood. Several cyclins have been identified with unique patterns of expression during spermatogenesis [1]. For example, the testis-specific A-type cyclin, cyclin A1, is restricted to spermatocytes during prophase I from the pachytene to diplotene stages [2]. Cyclin A1 was the first cyclin shown to be essential for meiosis: cyclin A1-deficient male mice are sterile due to an arrest in meiotic prophase at the diplotene stage, just prior to the first meiotic division [3]. In contrast, cyclin A2, which is generally considered to be the mammalian S-phase cyclin, is expressed in mitotically dividing spermatogonia but not in meiotic prophase spermatocytes [4]. Not surprisingly, deletion of the ubiquitously expressed cyclin A2 results in embryonic lethality shortly after implantation [5].

There are also two members of the mammalian E-type family, cyclin E1 and E2, which play important roles in mitotically-dividing cells. Cyclin E1 and E2 exhibit high homology within their protein sequence (70% identity between the cyclin box and 47% between the overall sequences) and it has been proposed that they have overlapping functions during the cell cycle. Indeed, *Ccne1* (to be designated as *E1* for simplicity in the rest of the text) or *Ccne2* (designated as *E2*) single knockout mice are viable but double-knockout mice die during embryonic development due to placental abnormalities [6], [7]. Interestingly, while both male and female *E1* knockout mice were fertile as were *E2* knockout females, *E2* knockout males exhibited reduced fertility, decreased testis size, reduced sperm counts and apparently abnormal meiotic spermatocytes [6]. However, neither the cellular nor molecular basis for this phenotype has been elucidated. Moreover, it is unknown which cells in the testis express the E-type cyclins, or the function that E1 and E2 might have in germ cells.

In the present study, we provide evidence for distinct functions of the E-type cyclins during spermatogenesis and novel regulation of their expression. We demonstrate that the E-type cyclins function in the progression of spermatocytes through meiotic prophase I, influencing homologous chromosome pairing, synapsis and DNA repair and, in particular, function at the chromosome ends. Further, in the absence of E-type cyclins, the proper localization of CDK2 on telomeres during male meiotic prophase I is disrupted and there is concomitant chromosome instability. These results reveal a critical role for the E-type cyclins during male mammalian meiosis and underscore their function in regulating spermatogenesis and hence, male fertility.

## Results

### Cyclins E1 and E2 have distinct patterns of expression during spermatogenesis

**Figure 1:**
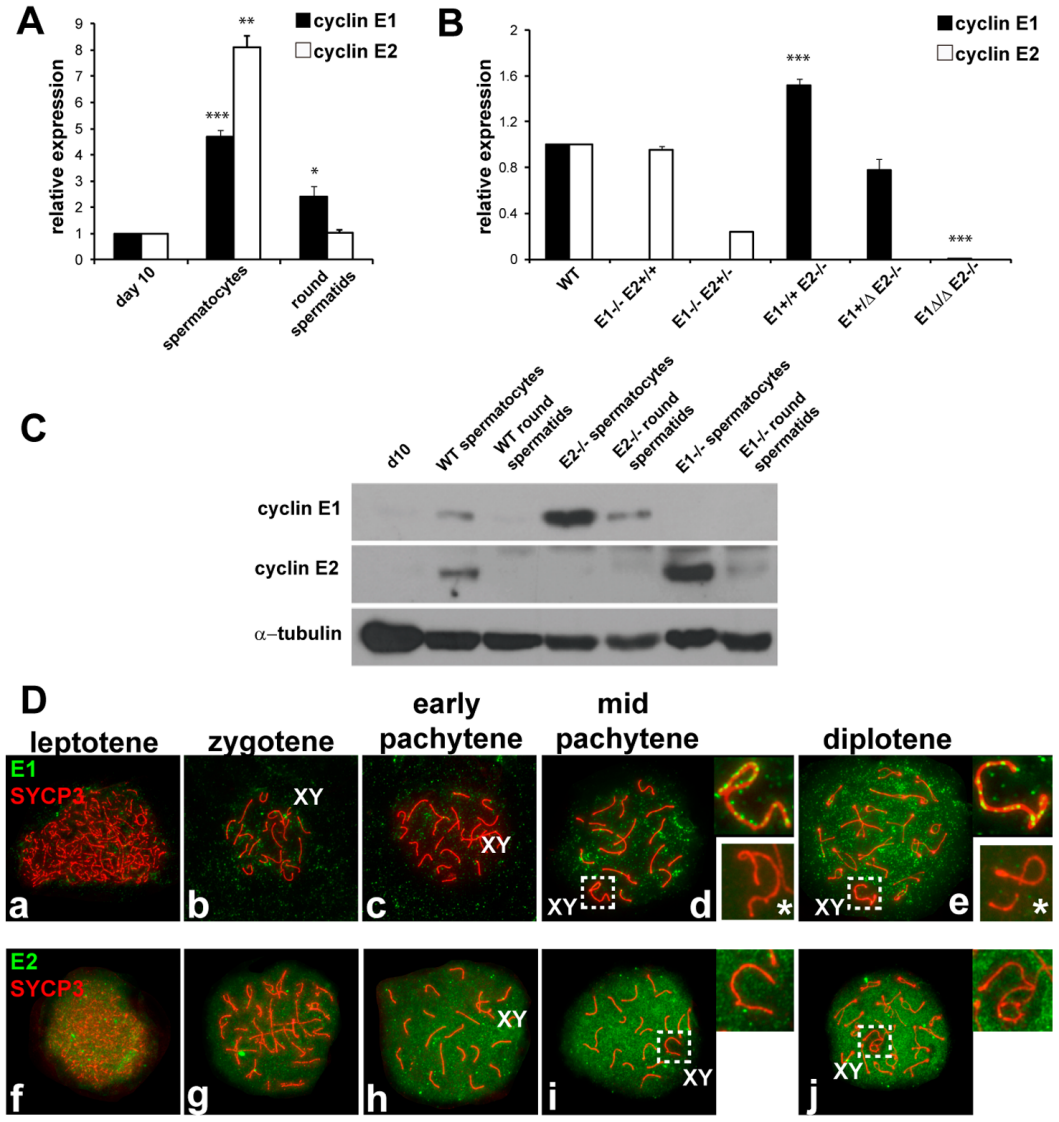
Cyclin E1 and E2 have differential mRNA and protein expression patterns during spermatogenesis. (A) Relative mRNA expression (compared to the expression of *Arbp* and normalized to expression levels obtained from pnd10 testes) was determined using RT-qPCR in pnd 10 testes and in purified populations of pachytene spermatocytes and round spermatids. Results are the mean ± SEM of four independent experiments. *p<0.05, **p<0.01, ***p<0.001. (B) Relative *E1* and *E2* mRNA expression (compared to the expression of *Arbp* and normalized to expression levels obtained from WT testes) using RT-qPCR of whole adult testes of different genotypes. Results are the mean ± SEM of three independent experiments. ***p<0.001. (C) Immunoblot analysis of E1 and E2 protein expression from tissues or purified populations of cells as noted. Immunodetection of α-tubulin was used as the loading control. (D) Localization of E1 (a–e) or E2 (f–j) (green) and SYCP3 (red) during prophase I in WT spermatocyte spreads. Right upper insets show the X and Y chromosomes (XY) outlined in d, e, i and j. E1 and E2 are totally absent in the XY of *E1−/−E2+/+* spermatocytes (lower right insets with asterisk in d,e).

To elucidate the role of E-type cyclins during spermatogenesis, we first identified their normal pattern of expression in the testis at the cellular and sub-cellular levels. Quantitative PCR analysis showed a robust *E1* mRNA expression in spermatocytes as compared to the mitotically dividing spermatogonia and Sertoli cells that predominate in post-natal day 10 testes [8] (Figure 1A). However, while E1 protein was readily detectable by immunohistochemistry in Sertoli cells in the adult testis, it was not evident in spermatocytes (Figure S1a,b). In fact, expression of E1 protein in spermatocytes was only detected in immunoblots of purified pachytene spermatocytes (Figure 1C) and in immuno-staining of chromosome spreads (Figure 1Da–e).

*E2* mRNA was also expressed in meiotic prophase spermatocytes (Figure 1A), but in contrast, E2 protein was consistently detected in spermatocytes by immunohistochemistry (Figure S1c–f), immunoblotting (Figure 1C), and chromosome spread (Figure 1Df–j) analyses. E2 protein was obviously detected in early (Figure S1f) to late pachytene spermatocytes (Figure S1f). It was also very faintly detected in dividing B-type spermatogonia (Bm, Figure S1e) and in preleptotene spermatocytes (PL, Figure S1d), but not in other spermatogonial stages (Figure 1C; Figure S1).

To precisely examine the developmental stage and sub-cellular localization of cyclin E1 and E2 proteins in meiotic prophase, we undertook a detailed immunolocalization analysis of spermatocyte spreads. Concomitant immunolocalization of SYCP3, a component of the axial element (AE) of the synaptonemal complex (SC), facilitated identification of meiotic chromosomes and classification of specific stages of prophase I. Cyclin E1 protein signal was barely detectable if at all in early pachytene spermatocytes, but was detected in mid-pachytene to diplotene spermatocytes in most of the chromatin (Figure 1Dd,e). However, in the sex chromosomes, the distribution of E1 was distinct, being present as foci along the AE (Figure 1Dd,e). The specificity of detection of E1 was confirmed by the absence of signal in spreads from *E1−/− E2+/+* testes (Figure 1Dd,e, lower right insets).

Cyclin E2 protein was clearly detected as early as the leptotene stage (Figure 1Df) and its expression increased throughout most of the chromatin during prophase I progression (Figure 1Dg–j). In contrast with E1, E2 was absent from the chromatin and AE of the X and Y (Figure 1Di,j). These results show clear differences between cyclin E1 and E2 proteins in their temporal appearance and levels of expression as well as their distinct nuclear distribution patterns during meiotic prophase.

### Altered cyclin E expression patterns in *E1−/−E2+/+* and *E1+/+E2−/−* testes

**Figure 2:**
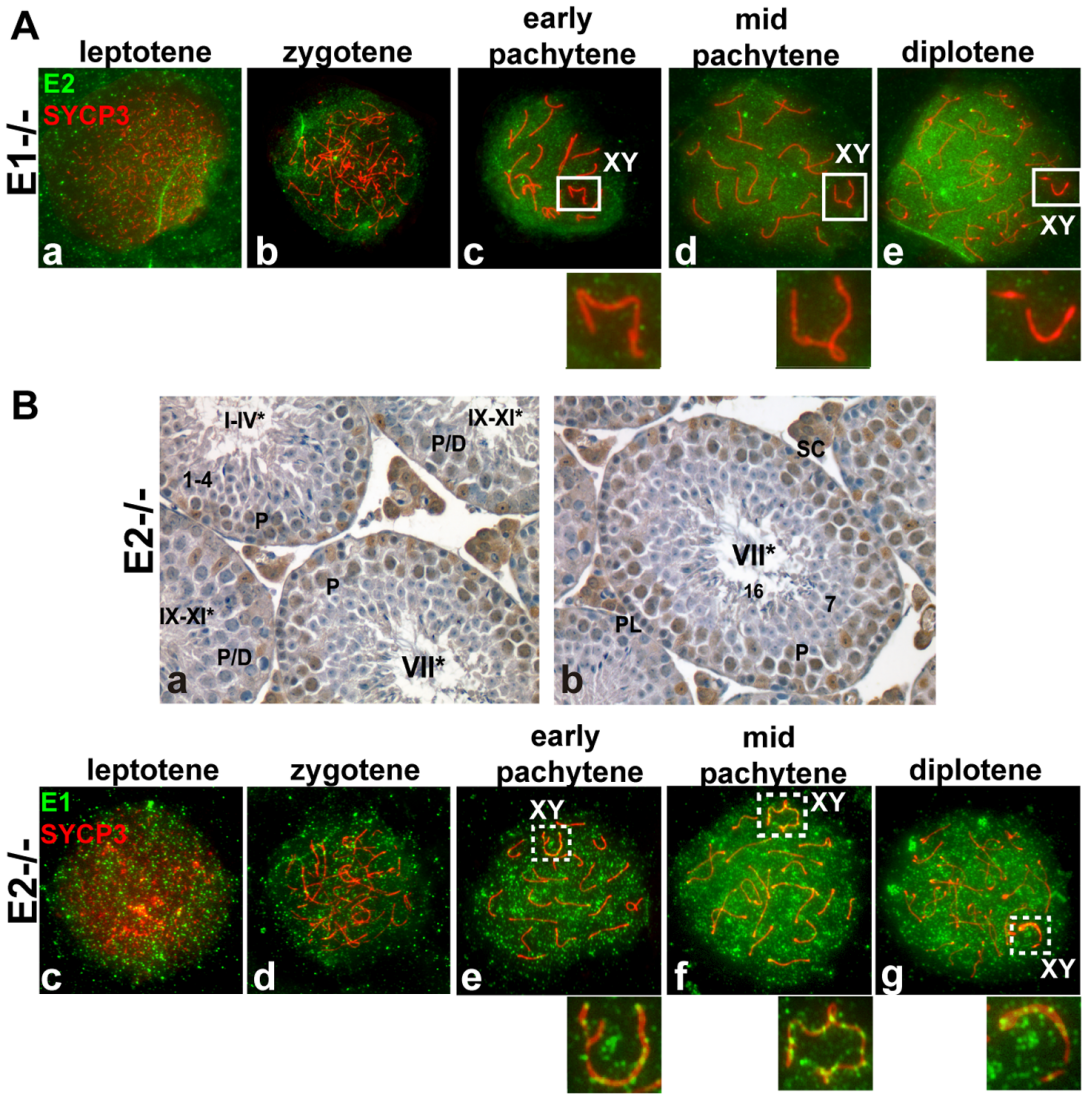
E1 levels and temporal pattern of expression are significantly altered in *E1+/+E2−/−* spermatocytes. (A) (a–e) Localization of cyclin E2 (green) and SYCP3 (red) during prophase I in *E1−/−E2+/+* spermatocytes. Insets magnify the sex chromosomes (XY) present in c–e. (B) (a,b) Immunohistochemistry of *E1+/+E2−/−* adult testes reveals the presence of E1 in Sertoli cells but also in pachytene and diplotene spermatocytes. Arabic numerals indicate step of spermatid differentiation; Roman numerals indicate stage of the tubules. (c–g) Localization of E1 (green) and SYCP3 (red) during prophase I in *E1+/+E2−/−* spermatocytes. E1 is now detected from leptotene to diplotene. E1 expression in the sex chromosomes (XY) remains as foci on the AEs (e–g, insets).

As functional redundancy between E1 and E2 had been suggested in mitotic cells [9] and both *E1−/−E2+/+* and *E1+/+E2−/−* mice are viable but E2-deficient male mice exhibit reduced fertility, we asked whether the pattern of expression of the remaining E type cyclin was altered, specifically in the testis. In *E1−/−E2+/+* testes, *E2* mRNA levels were not significantly changed (Figure 1B), but the levels of E2 protein were increased in spermatocytes and E2 protein was now also found in round spermatids (Figure 1C). However, the temporal appearance and distribution of E2 protein, including its exclusion from the X and Y, were unchanged in E1-deficient spermatocytes (Figure 2Aa–e).

In contrast, *E1* mRNA levels were increased (Figure 1B) and E1 protein was elevated in *E1+/+E2−/−* spermatocytes and was also detected in round spermatids by immunoblotting (Figure 1C). By immunohistochemistry, E1 protein was now also readily detected at low levels in early-pachytene spermatocytes (Figure 2Ba), increasing in mid-pachytene (Figure 2Ba,b), and was also detected in late-pachytene and diplotene spermatocytes (Figure 2Ba). No detectable levels of cyclin E1 protein were observed in spermatogonia (Figure 2Ba,b), preleptotene spermatocytes (Figure 2Bb) or round spermatids by immunohistochemistry (Figure 2Ba,b). In *E1+/+E2−/−* spermatocyte spreads, the temporal appearance of cyclin E1 protein now resembled that of cyclin E2, being detected as a faint signal in leptotene and increasing at zygotene (Figure 2Bc,d) and further increasing in pachytene and diplotene spermatocytes (Figure 2Be–g).

### Additional E-type cyclin deficiency causes progressive loss of advanced spermatogenic cells resulting in decreased fertility

We next examined the effects of additional deletions of E-type cyclin alleles on male fertility, using constitutive knockout *E1−/−E2+/+* and *E1+/+E2−/−* mice [6], conditional *E1* floxed mice [10], and mice expressing Cre under the *Stra8* promoter [11] (allele designated *E1Δ*). Mating studies of the resulting progeny with various combinations of deleted E-type cyclins showed that, as previously reported [6], [7], both *E1−/−E2+/+* and *E1−/−E2+/−* males were fertile (data not shown) but *E1+/+E2−/−* males exhibited reduced fertility. Detailed assessment of the nature of the reduced fertility revealed variable reduced sperm counts and variable numbers of fetuses/pups produced (Table S1A). Nonetheless, all *E1+/+E2−/−* males assessed (n = 10) were able to produce at least one fetus/pup. Importantly, the additional loss of one *E1* allele on the *E1+/+E2−/−* background had a striking effect on fertility: all *E1+/−E2−/−* males were completely infertile (n = 10), with a significant reduction in testis size (*p*<0.001) and complete azoospermia (Table S1B). As anticipated, the removal of the second *E1* allele in germ cells (*E1−/ΔE2−/−* or *E1Δ/ΔE2−/−*) also yielded sterile and azoospermic males.

**Figure 3:**
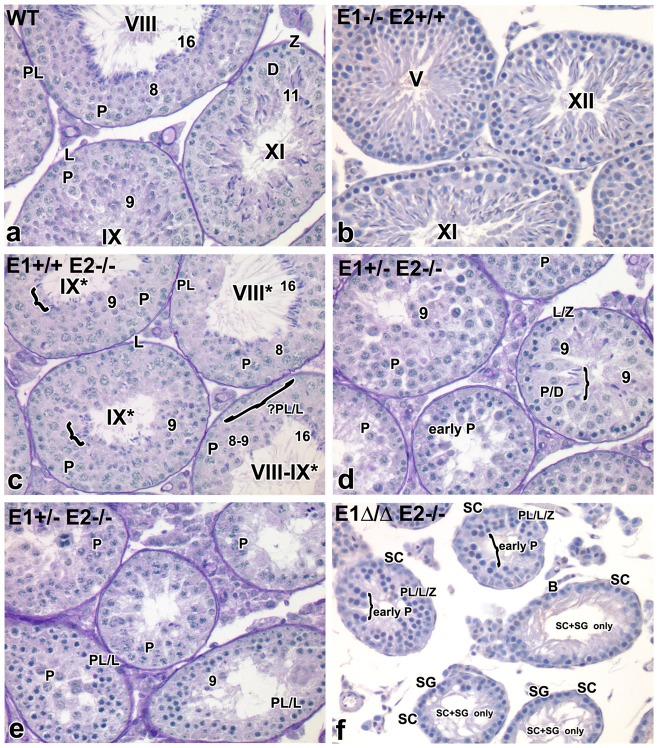
Additional E-type cyclin deficiency causes progressive loss of advanced spermatogenic cells. (a–f) Histological sections of representative seminiferous tubules in *E1−/−E2+/+*, *E1+/+E2−/−*, *E1+/−E2−/−* and *E1Δ/ΔE2−/−* testes counter-stained with PAS-hematoxylin. Magnification: a–f ×40. SC, Sertoli cells; PL/L, preleptotene-leptotene spermatocytes; Z, zygotene spermatocytes; D, diplotene spermatocytes; P, pachytene spermatocytes; RS, round spermatids. The missing layer of specific cell types (Figure 3c) is indicated with a bracket and question mark. Arabic numerals indicate step of spermatid differentiation; Roman numerals indicate stage of the tubules. Although abnormal cell associations complicate staging, an attempt was made using the acrosomal system [45], and tubules are labeled with a Roman numeral followed by an asterisk (*e.g.* stage IX*).

To begin to elucidate which spermatogenic cell types were affected by additional loss of E-type cyclin function, histological analysis of testes from adult mice of the various genotypes was performed (Figure 3a–f). *E1−/−E2+/+* and *E1−/−E2+/−* testes appeared morphologically normal (Figure 3b and data not shown, respectively), as compared to wild type (WT) testes (Figure 3a).

As shown in Figure 3c, *E1+/+E2−/−* testes displayed testicular abnormalities, as noted in earlier studies [6], but spermatogenesis was not arrested at a unique stage. The histological abnormalities became more severe with additional loss of *E1* alleles. That is, *E1+/−E2−/−* adult testes (and similarly *E1+/ΔE2−/−* testes) contained a few tubules with spermatogenesis arrested at the spermatid stage, but such spermatids were sparsely populated and mostly degenerating (Figure 3d,e). Abnormally elongated spermatids (Figure 3d, bracket) or a few step 9 spermatids were the most advanced spermatogenic cell types (Figure 3d). Most mice displayed very severely disrupted spermatogenesis, with tubules typically containing only pachytene spermatocytes (Figure 3e), while others contained preleptotene-leptotene spermatocytes with a few step 9 spermatids (Figure 3e).

Complete deletion of cyclin E function in the male germline (*E1Δ/−E2−/−* or *E1Δ/ΔE2−/−*) yielded profound disruption of spermatogenesis, with testicular tubules containing spermatocytes mostly arrested at early pachytene stages (Figure 3f). However, there were also some “Sertoli cell- and spermatogonia-only” tubules in adult testes (Figure 3f). It is interesting to note that most of the spermatogonia in these tubules are B-type, suggesting spermatogenesis may be delayed at the entry of B-type spermatogonia into preleptotene spermatocytes (Figure 3f).

### Additional cyclin E depletion increases apoptosis of pachytene cells

To determine whether the cells in the abnormal testicular tubules of the various genotypes were undergoing apoptosis, TUNEL staining of adult testicular sections was used. In WT adult testis, a few TUNEL-positive spermatogonia and early meiotically dividing spermatocytes can be seen (Figure S2a) [12]. In *E1−/−E2+/+* testes (Figure S2b), the pattern of TUNEL-positive germ cells was similar to that of WT. However, in *E1+/+E2−/−* testes (Figure S2c), TUNEL-positive pachytene spermatocytes were observed. Such TUNEL-positive cells were also detected in *E1+/−E2−/−* testes (Figure S2d), regardless of whether the testicular abnormalities (as reflected in the loss of advanced spermatogenic cells) were relatively modest (Figure 3d) or extensive in severity (Figure 3e). Given the greatly reduced cellularity in the *E1Δ/ΔE2−/−* tubules, the number of cells that could be detected actively undergoing apoptosis was comparatively low. However, as above, any detectable TUNEL-positive cells were apparently early pachytene spermatocytes (Figure S2e).

### E-type cyclin-deficient spermatocytes exhibit aberrant progression through prophase I

To evaluate the effects that additional depletion of E-type cyclin alleles produce in prophase I progression, we quantified the number of spermatocytes in each stage of prophase I among the various genotypes, identifying the respective stages by immunolocalization of SYCP3 to identify the AE of the SC, chromosome morphology, and the behavior of the X and Y [13]. Immunolocalization of SUMO-1 to the sex body served as a marker for the transition of early to mid-pachytene and late prophase stages [13].

The number of cells at various stages of prophase I were similar between *E1−/−E2+/+* and WT spermatocytes, with the highest proportion of cells being in the pachytene and diplotene stages (Figure S3a). In *E1+/+E2−/−* testes, there was a slight increase in the proportion of spermatocytes in earlier stages, such as leptotene through early pachytene (Figure S3b). Strikingly, in *E1+/ΔE2−/−* testes, we observed an increase in the proportion of spermatocytes in early stages of prophase I: most of the cells were in the zygotene and early pachytene stages with only 12.6±1.16% comprising the later stages as compared with control (Figure S3c). This suggests that spermatocytes accumulate at the zygotene/early pachytene stages, thus reducing the number of cells that can progress through later stages. This was even more apparent in *E1Δ/ΔE2−/−* testes, where most spermatocytes were in late zygotene or early pachytene and only 1.5±0.4% progressed into a mid pachytene-like stage, albeit highly aberrant (Figure S3d). No diplotene spermatocytes were observed.

### Cyclin E1-deficiency associates with altered synapsis of the sex chromosomes and E2-deficiency with defects in pairing and synapsis of autosomes

**Figure 4:**
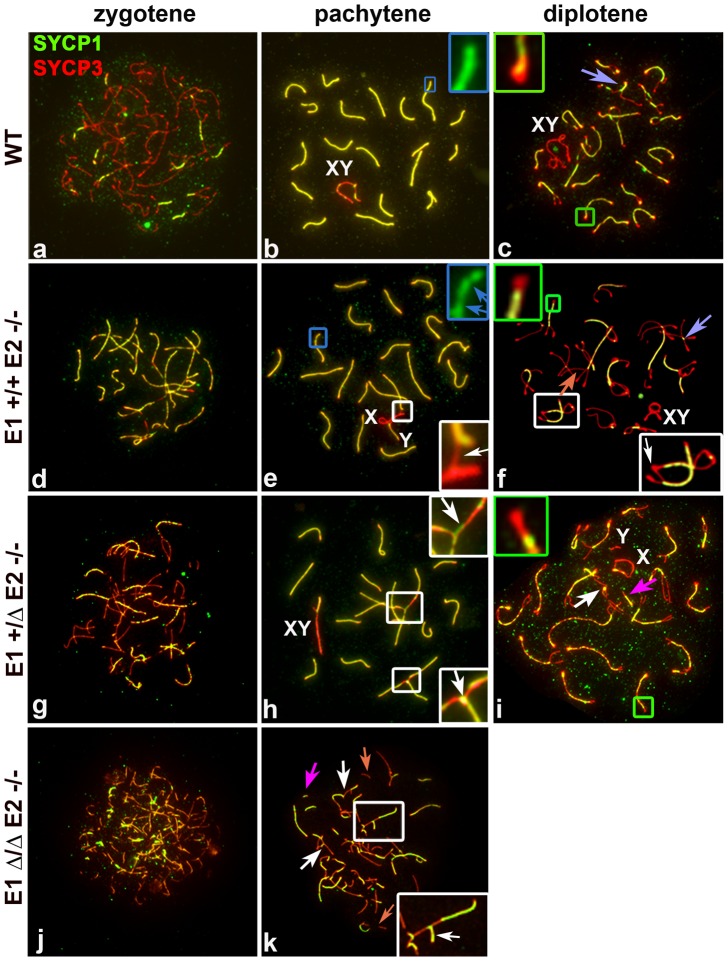
E2 deficiency disrupts normal pairing and synapsis that is exacerbated by loss of E1. Chromosome spreads from adult WT (a–c), *E1+/+E2−/−* (d–f) *E1+/ΔE2−/−* (g–i) and *E1Δ/ΔE2−/−* spermatocytes (j,k) immunostained with SYCP1 (green) and SYCP3 (red). Insets are a magnification of the outlined area. Schematic interpretations of the chromosomal associations shown in the insets are presented in Figure S5. (a–c) Normal progression of synapsis in WT spermatocytes. (b) Uniform SYCP1 pattern in the SC during pachytene (blue inset). (c) Chromosomes remain partially attached by chiasma (purple arrow) and SCAS are evident (green inset). (d–k) Irregular SYCP1 loading in *E1+/+E2−/−*, *E1+/ΔE2−/−* and *E1Δ/ΔE2−/−* spermatocytes at pachytene (e,blue inset, blue arrows; h,k) and diplotene (f,i); non-homologous chromosome associations (white insets) involving the XY chromosomes (XY) and formation of SC (white arrows). (i,k) Note the presence of short fragments of AE (light orange arrow) and SC (dark pink arrows). (i) Thinner SCAS than in WT chromosomes are visible at diplotene (green inset).

In mammalian meiosis, arrest at the pachytene stage and the subsequent induction of apoptosis are often triggered by abnormalities in homologous chromosome pairing and synapsis. We therefore analysed the pattern of chromosome pairing and synapsis in the different genotypes by immunolocalization of SYCP3 along with SYCP1, the main component of the central element (CE) of the SC. In normal meiosis, SYCP3 is initially loaded onto chromosomes during the leptotene stage and AE formation is completed in zygotene (Figure 4a). At this stage, the CE begins to form between the two AEs (Figure 4a) and SC formation is complete by the beginning of the pachytene stage (Figure 4b). At the diplotene stage, homologous axes separate and remain attached by chiasmata (Figure 4c). During later stages of prophase I, it is common to observe a conical thickening of the AEs at the chromosome ends called synaptonemal complex attachment sites (SCAS) (Figure 4c). The SCAS are crucial for attaching the chromosomes to the nuclear envelope [14].

In *E1*-deficient (*E1−/−E2+/+*) spermatocytes, the formation of the AEs and the loading of the CE (Figure S4a,b), as well as the structure of SCAS (data not shown), were similar to WT spermatocytes. Although fertility was not affected in these mice, we nonetheless observed some abnormalities in sex chromosome synapsis. Total asynapsis of the X and Y was seen in 25.5±2% of pachytene spermatocytes (*n* = 85) (Figure S4c). Of these asynapsed sex chromosomes, 8.2±1.7% exhibited the Y chromosome in self-synapsis or in a ring configuration (Figure S4a,e and d,f respectively). An additional 12±5.2% (*n* = 85) showed an incomplete synapsis of the pseudoautosomal region (PAR) of the X and Y that was restricted to a small portion of the sub-distal region of both chromosomes (Figure S4b). These results suggest that although the absence of cyclin E1 did not result in overtly impaired fertility, progression of synapsis of the PAR was altered.

In E2-deficient (*E1+/+E2−/−*) spermatocytes, wherein fertility was affected, spermatocytes progressed until the diplotene stage (Figure 4d–f). However, in some chromosomes of pachytene spermatocytes, the SC appeared interrupted. Although AEs were formed and aligned, the CE was not continuously assembled, as indicated by interrupted regions of SYCP1 (Figure 4e). These defects were observed in single or multiple chromosomes within the same cell and were detected in 22.8±2.7% of total pachytene spermatocytes (*n* = 100). Furthermore, we observed that 19±3.1% of pachytene spermatocytes had one or more chromosomes with heterologous associations that involved autosomes or autosomes with the X chromosome (Figure 4e). Almost all (96±1.4%) of these associations involved the telomeric ends in a “one to one” chromosome connection (Figure S5Ae) that persisted in diplotene spermatocytes (Figure 4f; S5Af), suggesting that spermatocytes with abnormal chromosome associations can progress through the pachytene stage. In addition, the SCAS at the chromosome ends were on average 12.5% reduced in length, compared to WT spermatocytes (Figure 4f; S5B).

The frequency of synapsis defects increased with loss of *E1* alleles in an E2-deficient background. In *E1+/ΔE2−/−* pachytene spermatocytes (Figure 4g–i), the frequency of intermittent SYCP1 localization on the SC increased to 54.9±7% (*n* = 57) and affected almost all the chromosomes. In *E1+/ΔE2−/−* testes, 29.6±4.1% (*n* = 57) of pachytene spermatocytes carried heterologous associations, which frequently involved chromosome ends with SYCP1 at the association sites (Figure 4h; S5Ah,h′). Notably, in *E1+/ΔE2−/−* spermatocytes, all chromosome ends exhibited thinner SCAS compared with WT chromosomes (on average, 25% reduced) (Figure 4i; S5B). The defects in AEs and SC formation were more severe in *E1Δ/ΔE2−/−* spermatocytes and were detected at earlier stages (Figure 4j,k). *E1Δ/ΔE2−/−* spermatocytes exhibited chromosome configurations that resembled those characteristics of leptotene and zygotene stages and a pachytene-like stage, but later stages of meiotic prophase were never observed. In the few spermatocytes that reached a pachytene-like stage, SYCP3 localization revealed small fragmented filaments that rarely formed continuous AEs. We also observed small fragments of SC and aberrant synapsis in the majority of the chromosomes (Figure 4k). In addition, most of the heterologous associated chromosomes formed complex chromosome chains (Figure 4k; S5Ak). In the very few *E1Δ/ΔE2−/−* spermatocytes observed in a mid-pachytene-like stage (characterized by the presence of SUMO-1 and γH2AX restricted to a defined region of the chromatin), the SCAS were 37.5% narrower compared with WT SCAS (Figure S5B).

### E-type cyclins are necessary for the normal progression of DNA double strand break (DSB) repair as well as for protecting chromosome ends

**Figure 5:**
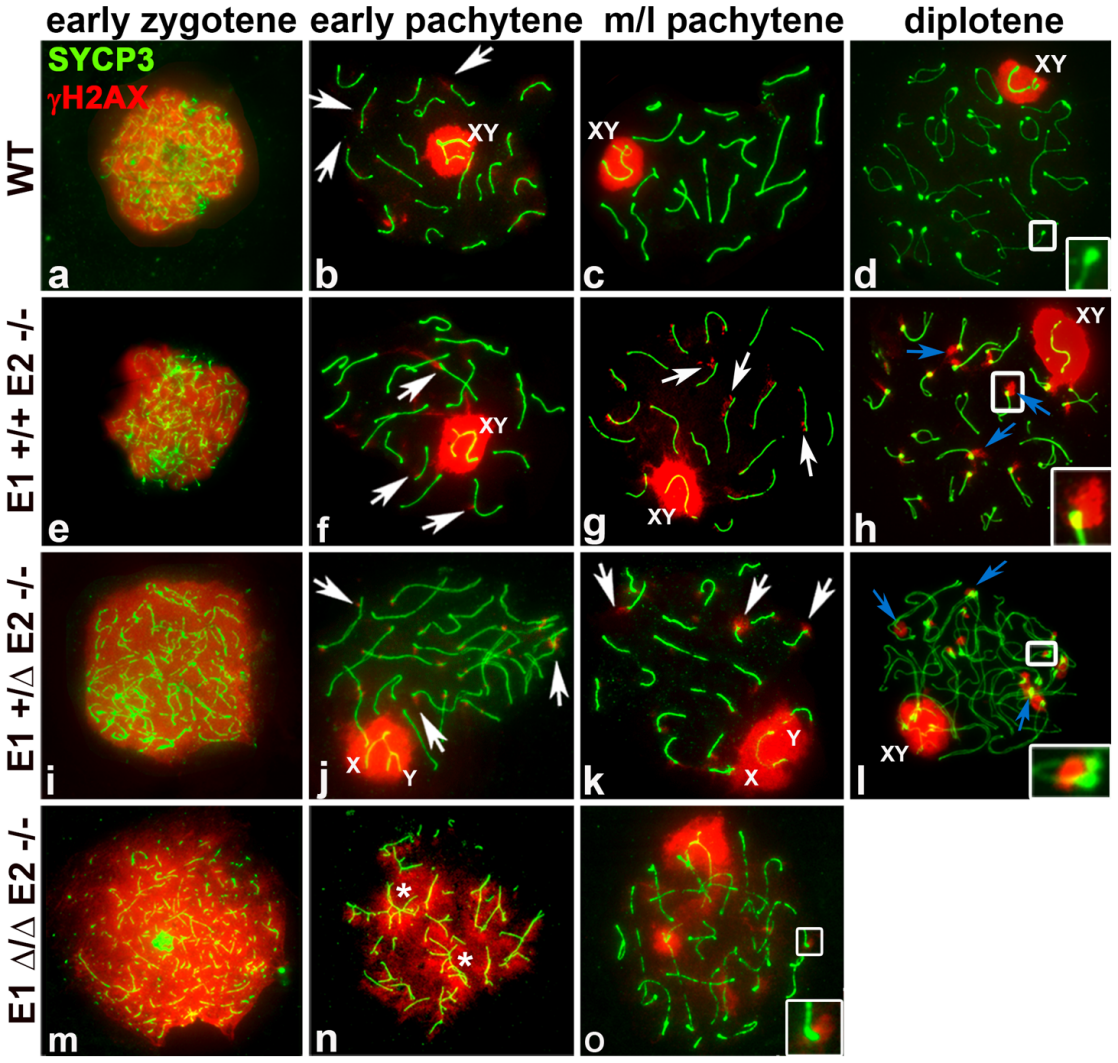
E2-deficient spermatocytes have unrepaired DSBs that are increased by depletion of E1. Chromosome spreads from adult WT (a–d), *E1+/+E2−/−* (e–h), *E1+/ΔE2−/−* (i–l) and *E1Δ/ΔE2−/−* spermatocytes (m–o) immunostained for γH2AX (red) and SYCP3 (green). Insets represent the area outlined. (a–d) γH2AX pattern in WT prophase I. (b) γH2AX localizes in the sex chromosomes and as foci in the chromatin adjacent to the SC in autosomes (white arrows). Telomeres in WT diplotene spermatocytes are devoid of γH2AX (d, white inset). (e–h) *E1+/+E2−/−* spermatocytes; (i–l) *E1+/ΔE2−/−* spermatocytes. γH2AX persists as foci in autosomes (g,j, white arrows) and in telomeres (g,k white arrows; h,l, blue arrows, insets). (m–o) γH2AX signal is distributed throughout the chromatin in *E1Δ/ΔE2−/−* early pachytene spermatocytes, primarily in unsynapsed chromosomes and chromosome associations (n, asterisks). The few spermatocytes observed at mid-pachytene displayed telomeric γH2AX foci (o, white inset).

To analyze whether the repair of DSBs was affected by depletion of E-type alleles, we analyzed spermatocyte spreads using γH2AX as a marker of DSBs [15]. WT leptotene-early zygotene spermatocytes exhibit γH2AX distributed throughout the entire nucleus (Figure 5a). In early pachytene spermatocytes, γH2AX was present only as small foci in the chromatin adjacent to the SC in the autosomes and throughout the chromatin of the X and Y (Figure 5b). In mid-pachytene to late diplotene spermatocytes, γH2AX was solely restricted to the XY body (Figure 5c,d).

In all the E-type cyclin-deficient genotypes, γH2AX was distributed throughout the chromatin during the leptotene-early zygotene stages, similar to WT spermatocytes (Figure 5e,i,m). In *E1−/−E2+/+* spermatocytes, the γH2AX pattern was similar to WT throughout prophase (Figure S4c). In contrast, in *E1+/+E2−/−* and *E1+/ΔE2−/−* early and mid-late pachytene spermatocytes, γH2AX was not only present in the sex chromosomes, but also persisted as foci in the chromatin adjacent to the SC in autosomes, particularly at the chromosome ends (Figure 5f,g,j,k). These telomeric foci were more prominent during the diplotene stages of E2-deficient spermatocytes (Figure 5h,l). Moreover, these defects were exacerbated upon loss of the remaining *E1* allele. In the few *E1Δ/ΔE2−/−* pachytene-like spermatocytes, all unsynapsed chromosomes (Figure 5n,o) had γH2AX signal in the chromatin (Figure 5n), indicating defects in DSB repair. Notably, the rare mid-pachytene-like spermatocytes that could be observed also contained chromosomes with γH2AX in the telomeric regions (Figure 5o).

### Meiotic sex chromosome inactivation (MSCI) is not overtly affected in the absence of the E-type cyclins

**Figure 6:**
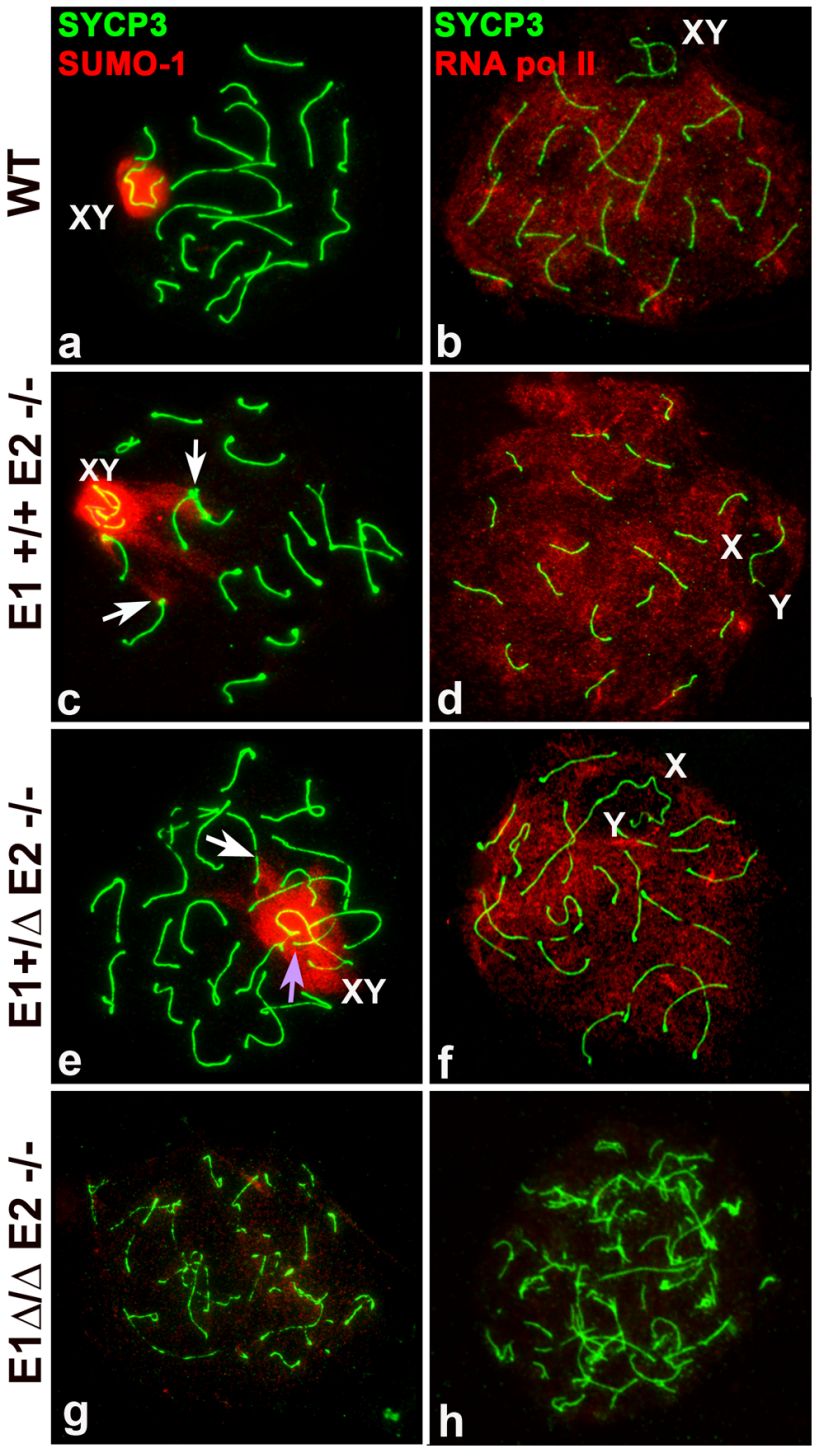
Meiotic sex chromosome inactivation appears unaffected by depletion of E-type cyclins. Chromosome spreads from adult wild type (WT) (a–b), *E1+/+E2−/−* (c–d), *E1+/ΔE2−/−* (e–f) and *E1Δ/ΔE2−/−* spermatocytes (g–h) immunostained for SUMO-1 (red) (a,c,e,g) or RNA pol II (red) (b,d,f,h) and SYCP3 (green). SUMO-1 is in the sex chromosomes (XY) in mid-pachytene to diplotene in WT (a), *E1+/+E2−/−* (c), and *E1+/ΔE2−/−* (e) spermatocytes. SUMO-1 also localizes in the telomeres and telomeric and subtelomeric regions of some chromosomes (c,e, white arrows) and in the chromatin of unsynapsed autosomes (e, purple arrow). (g) In most *E1Δ/ΔE2−/−* spermatocytes, SUMO-1 was absent from the chromatin. In *E1+/+E2−/−* (d) and *E1+/ΔE2−/−* (f) pachytene spermatocytes, RNA pol II is distributed throughout almost all the chromatin except where the sex chromosomes (X,Y) are localized, similar to WT (b). In *E1Δ/ΔE2−/−* testes, RNA pol II was not detected in most of the spermatocytes (h).

To begin to explore whether MSCI was compromised with loss of cyclin E function, we studied the pattern of distribution of SUMO-1, a marker of unsynapsed chromosomes [13], [16] and RNA pol II, a marker of transcriptional activity [13], [16]. In all the genotypes except where all E-type cyclin function is lost, SUMO-1 appeared in mid-pachytene to diplotene spermatocytes in the X and Y, similar to its distribution in WT spermatocytes (Figure 6a,c,e).

Interestingly, in *E1−/−E2+/+* mice, all (100%) of the mid/late pachytene spermatocytes expressed SUMO-1 in the X and Y, even if there was asynapsis in the PAR (Figure S4e). In *E1+/+E2−/−* and *E1+/ΔE2−/−* spermatocytes, SUMO-1 appeared not only in the sex chromosomes but also in unsynapsed autosomes (Figure 6c,e), indicating that asynapsis is properly recognized in mutant spermatocytes. That is, 93±4.2% of *E1+/+E2−/−* and 69±6.1% of *E1+/ΔE2−/−* pachytene spermatocytes (*n* = 80 and 67, respectively) had SUMO-1 in the sex chromosomes, indicating that the majority of spermatocytes of these genotypes were able to progress through mid/late pachytene. Interestingly, in *E1+/+E2−/−* and *E1+/ΔE2−/−* pachytene spermatocytes, we also observed SUMO-1 signal at the ends of a few chromosomes (Figure 6c,e). The complete absence of both E-type cyclins resulted in >98% of spermatocytes arresting in early prophase, thus, SUMO-1 was absent from the chromatin (Figure 6g). In the rare mid pachytene-like spermatocytes that were found in *E1Δ/ΔE2−/−* testes, a hint of SUMO-1 signal was observed but there was no clearly sex body formation (data not shown).

To determine whether the E-type cyclins were involved in transcriptional silencing of the sex chromosomes, we examined the general transcriptional status in spermatocytes by immunolocalization of RNA pol II in chromosome spreads. In WT spermatocytes, RNA pol II appeared at the beginning of the pachytene stage as a very low signal (data not shown) that increased in intensity as prophase I progressed. From mid-pachytene through diplotene, RNA pol II was detected as a bright signal distributed throughout almost all chromatin but was excluded from the sex body (Figure 6b), which reflects the transcriptional silencing of the sex chromosomes. This temporal and distribution pattern of RNA pol II was not affected by depletion of E1 (Figure S4d) or E2 (Figure 6d) nor in the spermatocytes that reached late prophase I stages in *E1+/ΔE2−/−* testes (Figure 6f). This suggests that depletion of E-type cyclins does not affect the transcriptional silencing of the sex chromosomes during prophase I. However, in the complete absence of both E-type cyclins, most of the spermatocytes never reach a pachytene-like stage and therefore, these nuclei lack RNA pol II signal (Figure 6h). In exceptional cases, although their chromosome morphology was completely altered, few spermatocytes exhibited low levels of RNA pol II in the chromatin (data not shown) but no recognizable sex body was formed.

### Deficiency of E-type cyclins results in abnormal telomere structure and telomere instability during prophase I

**Figure 7:**
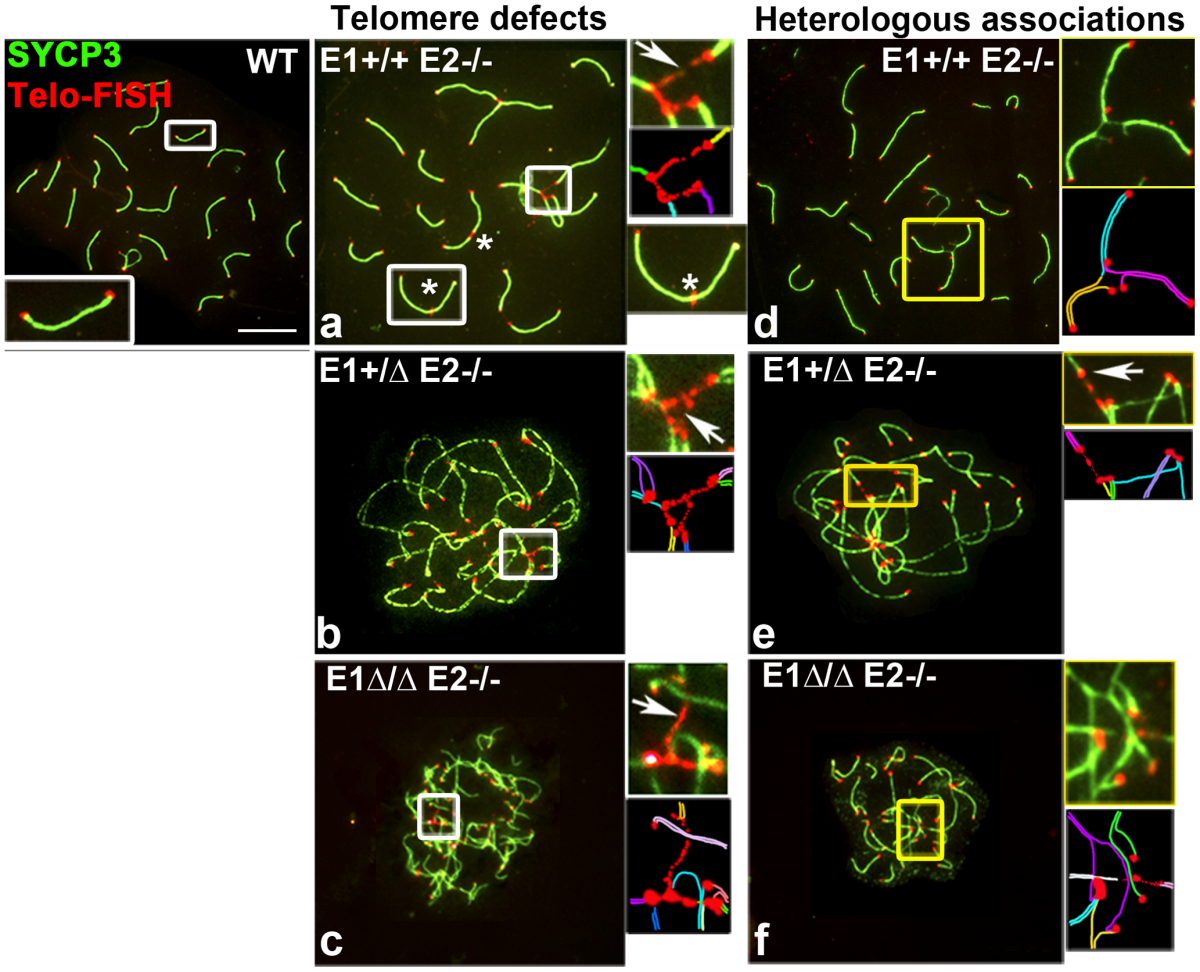
Telomere stability and chromosome integrity are increasingly disrupted by loss of E-type cyclin function. Chromosome spreads from adult WT, *E1+/+E2−/−* (a,d), *E1+/ΔE2−/−* (b,e) and *E1Δ/ΔE2−/−* pachytene spermatocytes (c,f) stained to detect telomeres (Telomere-FISH, red) and SYCP3 localization (green). Additional E-type cyclin deletion produces extended bridges between telomeres (a–c,e, white arrows and white insets), leading to telomeric fusions and chromosome rearrangements (asterisks): heterologous associations (d,e,f) and complex chromosome chains (d,e,f, yellow insets). A schematic representation of each inset is shown below the original, using one color for each chromosome (a–f).

The end-to-end heterologous chromosome associations and the presence of γH2AX foci at chromosome ends observed in mutant spermatocytes suggested that the telomeres might be abnormal. Indeed, immuno-FISH analyses on chromosome spreads revealed telomere defects that were never, or at most very rarely, present in WT chromosomes. The abnormalities included extended telomeres, heterologous telomere associations, and frequent telomere fusions in both autosomes and sex chromosomes (Figure 7), defects that have been associated with telomeric instability in other models [17]–[21]. In almost all *E1+/ΔE2−/−* and *E1Δ/ΔE2−/−* spermatocytes, we observed at least one chromosome with prominently extended telomere fibers, most commonly in association with other telomeres to form telomeric bridges between non-homologous chromosomes (Figure 7a–f). Moreover, ends of two or more individual SC were tightly associated and occasionally led to end-to-end fusions that produced chromosome rearrangements (Figure 7a). These defects increased with the additional depletion of *E2* and *E1* alleles, resulting in a frequency of 16.9±2.3%, 57.8±1.5% and 83.0±0.8% of bridges and/or telomere associations in *E1+/+E2−/−*, *E1+/ΔE2−/−* and *E1Δ/ΔE2−/−* pachytene spermatocytes, respectively (*n* = 50).

### E1 and E2 associate with CDK2 and are involved in its proper localization in meiotic prophase I spermatocytes

**Figure 8:**
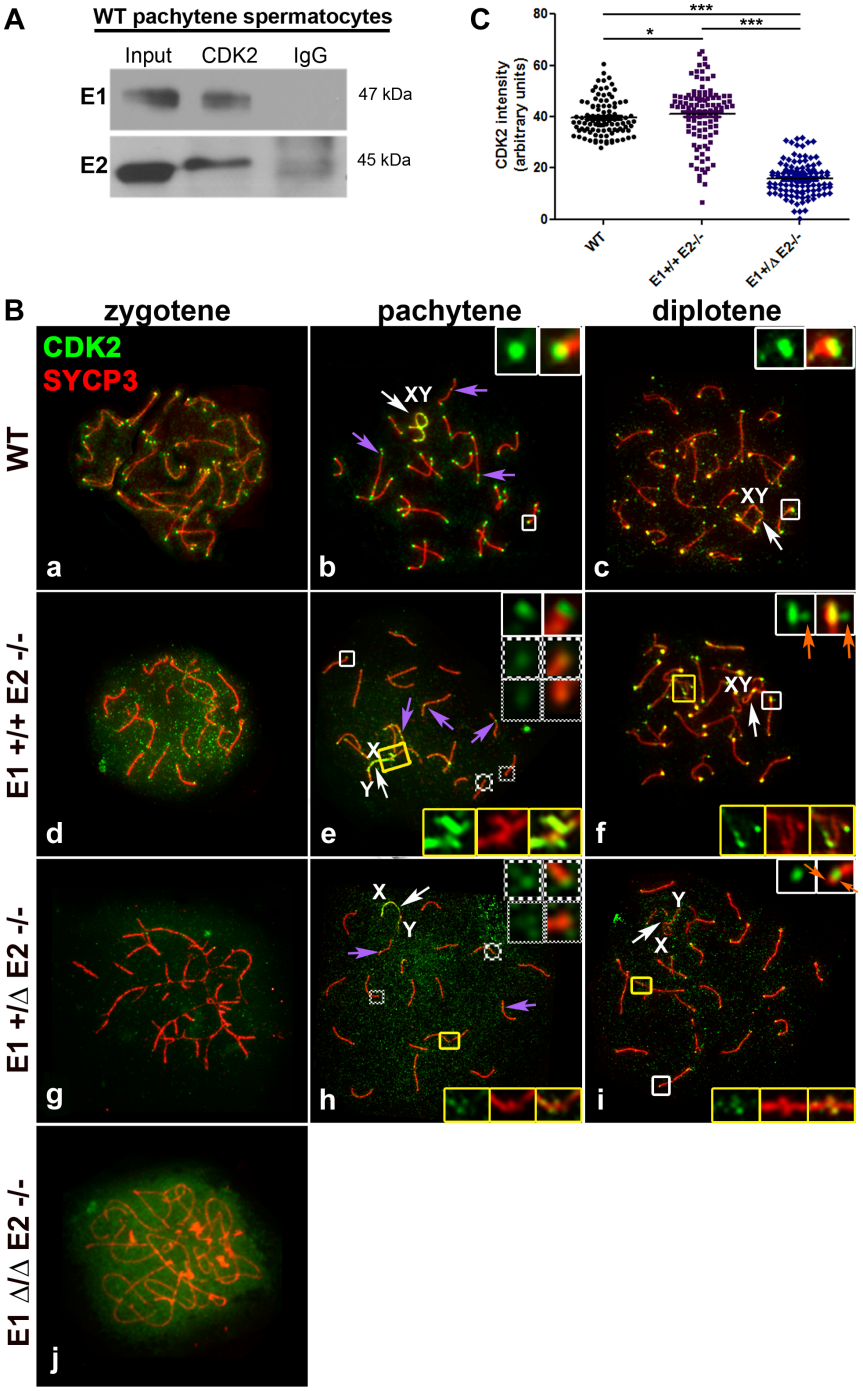
E1 and E2 associate with CDK2 and are crucial for its proper localization during male meiotic prophase I. (A) Coimmunoprecipitation of E1 or E2 with CDK2 from (WT) pachytene spermatocytes. (B) Chromosome spreads from adult WT (a–c), *E1+/+E2−/−* (d–f), *E1+/ΔE2−/−* (g–i) and *E1Δ/ΔE2−/−* spermatocytes (j), immunostained with anti-CDK2 (green) and anti-SYCP3 (red). In WT pachytene and diplotene spermatocytes, CDK2 localizes at the telomeres (b,c, white insets), late recombination nodules (LRN) and sex chromosomes (b, purple arrows and b–c, white arrows respectively). In *E1+/+E2−/−* spermatocytes, most telomeres presented a CDK2 signal similar to WT telomeres (e, white inset). However, CDK2 signal was reduced 1.6-fold in 28.2% and 2.5-fold in 12.81% of the telomeres respectively (e, solid-line and thinly-dotted insets, respectively). In diplotene spermatocytes, some telomeres exhibited a scattered CDK2 signal (f, white insets, orange arrows). CDK2 signal was also either scattered or lost in the chromosome ends involved in heterologous associations (e,f, yellow insets). In *E1+/ΔE2−/−* spermatocytes, CDK2 signal was also variably reduced in the telomeres (h, solid-line and thinly-dotted insets) and it was lost in the regions with heterologous associations (h,i, yellow insets). Localization of CDK2 at telomeres was also scattered in diplotene spermatocytes (i, white inset, orange arrows). Purple arrows in e and h indicate CDK2 localization in the LRNs. White arrows in e,f and h,i indicate the sex chromosomes (XY). In *E1Δ/ΔE2−/−* spermatocytes, CDK2 localizes diffusely throughout the nucleus (j). (C) Quantification of the intensity of CDK2 signal in telomeres of WT, *E1+/+E2−/−* and *E1+/ΔE2−/−* pachytene spermatocytes. *n* = 120 telomeres per each genotype. *p<0.05, ***p<0.001.

Cyclin E1 and E2 are known to interact with CDK2 in mitotic cells. We performed co-immunoprecipitation analysis of WT pachytene spermatocytes to confirm that both cyclin E1 and E2 indeed interacted physically with CDK2 in spermatocytes *in vivo* (Figure 8A). Interestingly, CDK2 has previously been shown to localize at telomeres, late recombination nodules (LRN), and in the sex body in prophase I spermatocytes [22] Therefore, we next examined CDK2 immunolocalization during prophase I in all E-deficient genotypes and performed a semi-quantitative analysis of the distribution of CDK2 localization. In WT spermatocytes, CDK2 first appeared in leptotene/zygotene spermatocytes (Figure 8Ba) as a faint signal at the telomeres. During the pachytene stage, CDK2 increased dramatically at the telomeres and was also observed in the LRN and in AEs of the X and Y (Figure 8Bb). At this stage, 90.8±6.5% of telomeres exhibited an average of 39.5 arbitrary units (au) (defined in Materials and Methods) of the intensity of CDK2 signal (n = 120 telomeres) (Figure 8C). At the diplotene stage, CDK2 was present in autosomal telomeres and was still associated with the X and Y chromosomes (Figure 8Bc). A similar pattern of CDK2 localization was observed in E1-deficient (*E1−/−E2+/+*) spermatocytes, interestingly, even when the X and Y were fully asynapsed (Figure S4f).

In E2-deficient spermatocytes (*E1+/+E2−/−*), however, the intensity of the telomeric signals from zygotene to diplotene spermatocytes were weaker compared to WT (Figure 8Bd–f), particularly during the pachytene stage, where telomeres exhibited a high heterogeneity in the intensity of CDK2 signal (Figure 8Be; 8C). Specifically, 59.0±9.6% of telomeres exhibited a CDK2 intensity similar to wild type spermatocytes _(_ *x* = 44.5 au) (Figure 8Be; 8C). However, 41.0±6% of telomeres exhibited lower intensities of CDK2 signal compared to WT (Figure 8Be; 8C). Occasionally, in individual chromosomes (Figure 8Bf) or chromosomes involved in an “end-to-end association” (Figure 8Be), CDK2 was diffusely distributed in the chromosome ends. This suggested that the loading of CDK2 upon telomeres was compromised in the absence of cyclin E2, but there were not significant alterations in the localization of CDK2 in the LRN and in the sex chromosomes, even when these chromosomes were aberrantly associated with other autosomes (Figure 8Be). That is, similar to WT spermatocytes, in *E1+/+E2−/−* spermatocytes, CDK2 localized mainly in the X chromosome and less intensely in the Y chromosome (Figure 8Be,f).

The defective localization of CDK2 in the telomeres became more severe with the additional depletion of *E1*. In *E1+/ΔE2−/−* spermatocytes, CDK2 signal was reduced in 92.7±6.2% of the telomeres (Figure 8Bh; 8C) and was almost completely absent from the ends of chromosomes that were associated or fused (Figure 8Bh,i). Similarly, weaker CDK2 signals in LRN and sex chromosomes were observed in *E1+/ΔE2−/−* spermatocytes (Figure 8Bh). The telomeric signal of CDK2 in *E1+/ΔE2−/−* diplotene spermatocytes also exhibited either a heterogeneous intensity or was not localized at the end of the chromosomes (Figure 8Bi), revealing that in the absence of cyclin E2, reduced levels of E1 had dramatic effects on CDK2 telomeric localization during prophase I. In the absence of all E-type cyclin proteins, the pattern of CDK2 localization was totally disrupted and only a diffuse signal throughout the nucleus was observed (Figure 8Bj).

## Discussion

In this study, we describe an essential role for the E-type cyclins in the regulation of mammalian male meiotic prophase I, controlling prophase I progression and regulating telomere and chromosome integrity. Surprisingly, expression of both E1 and E2 is not detected in most mitotic spermatogonia but is rather characteristic of meiotic spermatocytes and exhibits distinct expression patterns. E1 protein is expressed at low levels mainly in later stages of prophase I (pachytene and diplotene spermatocytes) while E2 protein can be detected as early as preleptotene, increasing throughout prophase I until the diplotene stage. When present, both E1 and E2 localize to the chromatin of autosomes and thus may co-localize in late prophase. However, localization in the chromatin of the XY body is strikingly different: E2 is never associated with the X or Y while E1 localizes as foci along the AEs of the sex chromosomes.

Co-expression of the two E-type cyclins has been widely observed in mitotic cells and it has been suggested that they exert overlapping functions during G1/S progression [9]. Support for this idea was obtained from the viability of both *E1−/−E2+/+* and *E1+/+E2−/−* single knockout mice and the lethality of *E1−/−E2−/−* mice [6], [7]. It has also been shown that cyclin *E2* depletion in the liver induces up-regulation of E1 expression at both the mRNA and protein levels and increases E1-CDK2 complex activity [23]. We found that depletion of E1 or E2 protein in spermatocytes induces an upregulation of E2 or E1 protein, respectively. This potential compensatory mechanism is most striking in the increase in levels of mRNA and protein expression of E1 upon E2 depletion and the ectopic presence of E1 protein in meiotic stages where E2 is normally expressed. However, the elevated levels of E1 do not fully compensate for loss of E2, as *E1+/+E2−/−* mice exhibit reduced fertility. This could be due to the changes in E1 protein expression incompletely mimicking normal E2 expression during prophase I. The normal presence of low levels of E2 in preleptotene cells and dividing B-type spermatogonia raises the possibility of a pre-meiotic function as well, which could contribute to the early meiotic defects. However, although depletion of E2 induces the expression of E1 in earlier stages of prophase I, it did not alter the expression pattern of E1 in non-meiotic cells, lessening the likelihood of an important pre-meiotic function for the E-type cyclins.

Alternatively, and not mutually exclusively, the different expression pattern that cyclin E1 and E2 have during prophase I may hint to distinct functions for the two E cyclins during meiosis. Indeed, while loss of *E2* in the germline results in abnormal synapsis, heterologous chromosome associations, defects in CDK2 localization, and late γH2AX foci on autosomes, there is increased severity of the spermatogenic defects and complete sterility upon additional deletion of *E1* alleles, suggesting that E1 must have important functions as well.

Loss of cyclin E1 function principally affected synapsis of the PAR and structural modifications that occur in the AEs of the sex chromosomes. Although these defects were noticeable in only a subset of pachytene spermatocytes, the defective synapsis of the PAR could account in part for the increased severity in meiotic abnormalities and enhanced apoptosis upon additional loss of *E1* alleles on an E2-deficient background. Such pairing and synapsis defects of the PAR, as seen in spermatocytes that specifically lack the Spo11α isoform (but containing Spo11β) were proposed to trigger the spindle checkpoint during metaphase followed by apoptosis [24]. Based on the localization of E1 in the AEs of the X and Y and the pairing defects produced by its depletion, it appears that E1 could be involved in maintaining the stabilization of the PAR synapsis during the pachytene stage.

The interdependence between pairing, synapsis and DNA repair during mammalian meiosis makes it difficult to discriminate specifically which (or all) processes are affected by E2-deficiency and further reduction of E1 protein. Alternatively, it is possible that they are secondary effects of the disruption of other processes, such as telomere anchoring in the nuclear envelope and chromosome movement. Regardless of the underlying mechanism, pairing and synapsis appear to be more compromised than DNA repair in *E1+/+E2−/−* and *E1+/−E2−/−* spermatocytes. That is, at the leptotene stage, chromatin-wide γH2AX staining appeared normal in all mutant spermatocytes, suggesting that generation of DSBs is not affected by depletion of E1 and/or E2. Furthermore, most of the γH2AX foci disappear during the zygotene stage similar to WT spermatocytes, suggesting that most DSB repair is not compromised in *E1* and *E2* mutant spermatocytes. However, a few γH2AX foci remain in the chromatin adjacent to the AEs and, more obviously, in the telomeric/subtelomeric regions during late prophase. This implies that a DNA damage signaling is occurring at chromosome ends and that telomere integrity is affected by loss of E-type cyclins.

Among the more striking aspects of the phenotypes exhibited by the various E-type cyclin knockouts were the defective localization of CDK2 in telomeres, the concomitant loss of telomere structural integrity, and the presence of frequent telomere fusions, all of which increased with further loss of E1 alleles. Although neither cyclin E2 nor E1 was located specifically at the telomeres, we propose that formation of cyclin E-CDK2 complexes is necessary for the localization of CDK2 to the telomeres and the subsequent protection of the telomere ends. In support of this model, it should be recalled that CDK2-deficient spermatocytes have similar, but not identical, meiotic phenotype to *E1Δ/ΔE2−/−* spermatocytes [25], [26]. That is, in absence of CDK2, spermatocytes also exhibited abnormal chromosome rearrangements, non-homologous pairing and defective telomeres that were not attached to the nuclear envelope. However, *E1Δ/ΔE2−/−* spermatocytes exhibited a more severe phenotype in terms of the pairing and synapsis defects and spermatocyte progression throughout prophase I. It was proposed that CDK2 may play a role in the proper telomere dynamics during prophase I [26]; herein we further propose that E-type cyclins are likely regulating the telomere-specific activity of CDK2. Alternatively, and not mutually exclusively, it is also possible that the E-type cyclins can exert a function in telomere protection in a CDK-independent manner. Such kinase-independent functions have been previously demonstrated for cyclin E during G(0)/S phase progression [27], and for cyclin D during regulation of cell growth and cancer [28] and has been suggested for cyclin B3 during spermatogenesis [29].

Loss of CDK2 localization and activity at the telomeres could trigger the loss of telomere positioning and function, which in turn can explain in part the defects observed in chromosome pairing and synapsis exhibited by E-type cyclin mutant spermatocytes. That is, loss of telomere end protection could affect their proper anchoring to the nuclear envelope, an event that is fundamental for accurate pairing and synapsis of the chromosomes [30]. Thus, depletion of E cyclins could affect telomeric anchoring to and movement through the nuclear envelope and subsequently trigger the meiotic defects in the mutant spermatocytes. Similar pairing and synapsis defects were observed when telomeres are unprotected, as in SMC1β-deficient spermatocytes [31], or when telomere dynamics and anchoring are altered, as seen in spermatocytes lacking LMNA, SUN1, and SUN2 [32]–[34].

The presence of telomeric bridges between different chromosomes, together with the appearance of γH2AX and the generation of chromosome fusions are indicative of dysfunctional telomeres and thus, telomeric instability [17]–[21], [35]. Therefore, our results showed that E-type cyclins are required for normal telomere and chromosome stability during male meiosis and suggested that telomere homeostasis (i.e. telomere length and capping) are severely compromised [36]. Indeed, telomere uncapping could also explain the presence of γH2AX foci in the telomeric/subtelomeric regions and the thin SCAS observed in *E1Δ/ΔE2−/−* spermatocytes. That is, the inability to form functional cyclin E2- and E1-CDK2 complexes could result in telomere uncapping that could trigger an abnormal DNA damage checkpoint response, as demonstrated by the presence of γH2AX foci. Thus, possible targets of cyclin E-CDK2 complexes could also include proteins involved in telomere protection [37].

In summary, our findings indicate a critical requirement for cyclin E function in meiosis rather than mitosis in the male germline. The meiotic defects that are observed highlight E-type cyclins as essential regulators of male meiosis and strongly point to a mechanistic role for E-type cyclins in the maintenance of telomere integrity. The observations further provide evidence for distinct functions of the mammalian E-type cyclins, interestingly, in non-classical cell cycle regulatory events.

## Materials and Methods

### Generation of *E1−/−E2+/+*, *E1−/−E2+/−*, *E1+/+E2−/−*, *E1+/−E2−/−*, *E1+/ΔE2−/−* and *Cre-Stra8-E1flox/flox-E2−/− (E1Δ/ΔE2−/−)* male mice

All experiments involving mice were approved by the Columbia University Institutional Animal Care and Use Committee and performed in accordance with the National Institutes of Health guidelines for the care and use of animals. *E1+/−E2+/−* [6] mice were mated to obtain *E1−/−E2+/+*, *E1−/−E2+/−*, *E1+/+E2−/−* and *E1+/−E2−/−* male mice. *E1flox/flox* mice were generated as described [10]. *E1flox/flox* mice were interbred with *Stra8-Cre* mice [11] (expressing the Cre recombinase only in males beginning at postnatal day (pnd) 3 in early-stage spermatogonia through preleptotene-stage spermatocytes) then with *E1+/+E2−/−* mice or *E1+/−E2−/−* female mice to generate *E1Δ/ΔE2−/−* and *E1Δ/−E2−/−* male mice (lacking both E-type cyclins in the germ cell line, starting from early stage spermatogonia). As no differences were observed between *E1+/ΔE2−/−* and *E1+/−E2−/−*; and *E1Δ/ΔE2−/−* and *E1−/−E2−/*, we utilized alternately both genotypes in all experiments.

### Fertility studies

For each genotype (*E1−/−E2+/+; E1+/+E2−/−; E1+/−E2−/−*), males (n = 10) at 8 weeks were mated with two 8 week-old WT females for one consecutive month [38]. After this period the females were sacrificed and the number of fetuses and pups in the cage was quantified. Another two females were mated with the same male. This experiment was conducted three times in a row, after which, testes from euthanized males were dissected and weighed. Sperm counts were quantified from the caudal epididymis, as previously described [39].

### Quantitative PCR

One microgram of total RNA, isolated from cell separation samples or whole testes, using TRIZOL reagent (Invitrogen), was subjected to RT-qPCR, according to our standard protocol [40]. Primer sets are provided in Supplemental Experimental Procedures. The *acidic ribosomal phosphoprotein P0* (*Arbp*) gene was used as an internal control for data normalization.

Specific primers were designed as follows:

*E1* forward: 5′ GTGGCTCCGACCTTTCAGTC 3′

*E1* reverse: 5′ CACAGTCTTGTCAATCTTGGCA 3′

*E2* forward: 5′ AGGAATCAGCCCTTGCATTATC 3′

*E2* reverse: 5′ CCCAGCTTAAATCTGGCAGAG 3′

*Arbp* forward: 5′ CAAAGCTGAAGCAAAGGAAGAG 3′

*Arbp* reverse: 5′ AATTAAGCAGGCTGACTTGGTTG 3′

### Statistical analyses

Results represent mean ± SEM from at least three independent experiments. Statistical analyses between two parameters were performed using a non-parametric Mann Whitney U test (Prism4, Graphpad Software, Inc., San Diego, CA). The threshold of significance was set at 0.05.

### Cell separation, immunoblot

Preparation of enriched populations of pachytene spermatocytes and round spermatids was carried out according to our laboratory's established protocol [41], [42]. Purity of cell populations was assessed by flow cytometric analysis on a Becton Dickinson FACScan Flow Cytometer. Results were analyzed using CellQuest Pro software. Proteins were extracted from purified cell populations from adult testes as previously described [43]. Rabbit anti-cyclin E2 1∶500 (Abcam, ab32103), rabbit anti-cyclin E1 1∶3000 (provided by Dr. Jim Roberts, Fred Hutchinson Cancer Research Center) and mouse anti-α tubulin 1∶5000 (Sigma T6199) were used for immunoblot analysis according to our standard procedures [43].

### Co-immunoprecipitation

Cell lysates were prepared from adult WT testes as previously described [43]. Lysates were pre-cleared with protein A agarose beads (Roche, cat#11134515001) at 4°C for 1 h. Pre-cleared lysates were then incubated with mouse anti-CDK2 1∶30 (D-12) (Santa Cruz sc-6248) or IgG control for 4 h with gentle agitation at 4°C. Protein A agarose beads were added and incubated overnight. The beads and immunoprecipitated complexes were pelleted by a 10 s centrifugation at 500 g, and then washed in wash buffer (20 mM Tris, pH 8.0, 150 mM NaCl, 0.1% NP40) four times at 4°C. Final pellets were resuspended in 1XSDS loading buffer and boiled for 5 min. The supernatant was run on 10% SDS–PAGE and immunoblotting was performed as described above using antibodies specific for cyclin E1 and cyclin E2. Clean-Blot IP detection reagent HRP (Thermo Scientific, cat# 21230) was used as the secondary antibody at 1∶500.

### Immunohistochemistry, immunofluorescence (IF), immuno-FISH and image quantifications

For histology and immunohistochemistry, testes from different mice genotypes were dissected, fixed in Bouin's solution or 4% paraformaldehyde, respectively, and paraffin embedded, as previously described [44]. Primary antibodies against E1 or E2 were used at dilutions of 1∶100 and 1∶125, respectively.

Spermatocyte spreads and immunostaining were prepared as previously described [13]. For immunostaining, we used the following primary antibodies: rabbit anti-cyclin E2 1∶70 (Abcam, ab32103), rabbit anti-cyclin E1 1∶30 (kindly provided by Dr. Jim Roberts), mouse anti-SYCP1 1∶100 (Abcam ab15087), rabbit anti-SYCP3 1∶200 (Abcam, ab15093), mouse anti-phosphor-H2AX (Ser139) 1∶1000 clone JBW301 (Upstate 05-636), mouse anti-SUMO-1 1∶50 (Santa Cruz Biotechnology, sc-5308), mouse anti-CDK2 1∶30 (D-12) (Santa Cruz sc-6248) and mouse anti-RNA pol II CTD4H8 (Upstate 05-623). All secondary antibodies were diluted to 1∶200 in PBS: FITC-conjugated donkey anti-rabbit IgG (H+L), FITC-conjugated donkey anti-mouse IgG (H+L), TR-conjugated donkey anti-mouse IgG (H+L), DyLight 594 goat anti-rabbit IgG (H+L), DyLight 594 goat anti-rabbit IgG F(ab′)_2_, DyLight FITC goat anti-rabbit IgG, F(ab′)_2_, Alexa Fluor 350 donkey anti-mouse IgG, Alexa Fluor 350 donkey anti-rabbit IgG, FITC-conjugated donkey anti-goat IgG. Slides were counter-stained with DAPI and mounted with Vectashield (Vector Labs).

For combined immuno-FISH, we first performed immunofluorescence (IF) on chromosome spreads followed by telomere FISH. After counter-staining with DAPI and PBS rinsing, slides were incubated in 2X sodium saline citrate (SSC) 15 min at room temperature (RT). Slides were then dehydrated and air-dried. The slides were then denatured in 75% formamide/2X SSC at 85°C for 7 min, dehydrated in an ethanol series at 4°C and incubated with a Human Chromosome Pan-Telomeric probe (1696-CY3-01, CAMBIO, UK) overnight at 37°C. Finally, we performed three washes at 42°C (3 washes with 50% formamide/2XSSC and 3 washes in 2X SSC) followed by three washes in 4X SSC/0.1% Tween-20.

For light microscopy, observations were made in a Nikon Eclipse E800 using a 20X/NA: 0.5 or 40X/NA:0.75 Plan Fluor objective, equipped with a RTtm KE color 3-shot digital camera. Photographs were taken using Spot Advance software. For immunofluorescence, observations were made in a Nikon Eclipse 80i using a 100X/NA: 1.4 oil immersion objective, equipped with a QImaging Retiga EXi Fast 1394 digital camera. Images were captured with QCapture Pro software. All images were processed using Adobe Photoshop CS5 software.

SCAS length was quantified by measuring the width of each chromosome end in spermatocyte spreads, using the length measurement plugin in ImageJ (NIH). The images used for the measurements were improved using an inverted LUT to avoid potential pitfalls at the border of the SCAS. ANOVA and t-test were used to determine the differences between the values and the threshold of significance was set at 0.05.

CDK2 intensity was quantified by selecting the area of CDK2 signal in each telomere and measuring the intensity of the signal using the measurement/mean value tool in ImageJ (NIH). Both CDK2 signal and background were measured and the final CDK2 intensity was calculated by subtracting the background from the CDK2 signal. Two tailed t-test was used to determine the differences between the genotypes and the threshold of significance was set at 0.05.

### Terminal deoxynucleotidyltransferase-mediated deoxy-UTP nick end labelling (TUNEL) staining

TUNEL staining was performed on tissue sections using the *in situ* cell death detection kit (Roche Diagnostics, Indianapolis, IN) as previously described [12]. Only clearly stained cells were considered as apoptotic and only tubules cut perpendicular to the length of the tubule (round tubules in section) were evaluated.

## Supporting Information

Figure S1 Cyclin E2 protein but not E1 was consistently found in pachytene spermatocytes at all stages. Histological sections of testes from adult wild type (WT) were immunostained with anti-cyclin E1 (a,b) and anti-cyclin E2 (c–f) antibodies. Magnification: a,c ×20; b, d–f ×40. B, B-type spermatogonia; Bm, dividing B-type spermatogonia; PL, preleptotene spermatocytes; L, leptotene spermatocytes; Z, zygotene spermatocytes; D, diplotene spermatocytes; P, pachytene spermatocytes. Arabic numerals indicate the step of spermatid differentiation; Roman numerals indicate the stage of the tubules.
(TIF)

Figure S2 TUNEL-positive pachytene spermatocytes were found in the E-type cyclin deficient germline. Representative stages of seminiferous tubules containing TUNEL positive cells in wild type (WT, a), *E1−/−E2+/+* (b), *E1+/+E2−/−* (c), *E1+/−E2−/−* (d) and *E1Δ/ΔE2−/−* (e) testes. The most striking observation was the presence of TUNEL-positive pachytene spermatocytes in both *E1+/+E2−/−* and *E1+/−E2−/−* testes (c,d), regardless of the severity of the testicular abnormalities (as reflected from the loss of advanced spermatogenic cells). In addition, TUNEL-positive spermatids were not detected. Magnification: a–e ×40. PL/L/Z, preleptotene-leptotene-zygotene spermatocytes; P, pachytene spermatocytes, early P, early pachytene spermatocytes; mid-P, mid pachytene spermatocytes; RS, round spermatids. Arabic numerals indicate the step of spermatid differentiation; Roman numerals indicate the stage of the tubules.
(TIF)

Figure S3 Percentage of spermatocytes present in each stage of prophase I. Wild type (WT) (white bars) and mutant (black bars) spermatocytes: *E1−/−E2+/+* (a), *E1+/+E2−/−* (b), *E1+/ΔE2−/−* (c) and *E1Δ/ΔE2−/−* (d) spermatocytes. Each bar represents the mean number of spermatocytes obtained from one testis each from three mice per genotype. Per animal, a total of 400 (in *E1+/ΔE2−/−* and *E1Δ/ΔE2−/−* testis) and 500 spermatocytes (in all other genotypes) were counted. Error bars represent SEM.
(TIF)

Figure S4 E1 depletion solely disrupts the synapsis of sex chromosomes. Chromosome spreads from *E1−/−E2+/+* spermatocytes immunostained with SYCP3 (red) and SYCP1 (a–b); γH2AX (c), RNA pol2 (d), SUMO-1 (e) and CDK2 (f) (green). Insets represent the magnifications of the area selected in (a–f) (white squares) above their schematic representations. The X and Y chromosomes were frequently observed in total asynapsis (a,c,,d,f, insets) or in a peculiar synapsis that comprised only a small area in the pseudo-autosomal region (PAR) (b, green arrow). Y chromosome self-synapsis (insets in a,e, white arrows) or telomeres of the X or Y chromosome close together in a ring configuration (insets in c,e,f,) were observed.
(TIF)

Figure S5 A) Schematic representations of white insets shown in Figure 4 and B) SCAS measurements. e) Insets and their respective schemes of Figure 4e showing the association of an autosomal end with the X chromosome. f) Inset and schematic of Figure 4f. Two heterologous autosomes are associated through their chromosome ends (white arrow). h–h′) Insets and schematics of Figure 4h. Three heterologous autosomes are associated through their chromosome end. k) Inset and schematic of Figure 4k. Two heterologous autosomes are partially synapsed. Each color represents a different chromosome in the insets. **B**) SCAS measurements. *** *p*≤0.001, *n* = 6 cells per genotype.
(TIF)

Table S1 E2 depletion results in variable reduced sperm counts and numbers of fetuses/pups. *E1+/+E2−/−* (A), *E1+/ΔE2−/−* (B). *n* = 10 for both genotypes.
(PDF)
