# Supplementary figures and images for: Mammalian E-type Cyclins Control Chromosome Pairing, Telomere Stability and CDK2 Localization in Male Meiosis
Source: PLoS Genet. 2014 Feb 27;10(2):e1004165. doi: 10.1371/journal.pgen.1004165 (PMC3937215; doi:10.1371/journal.pgen.1004165)

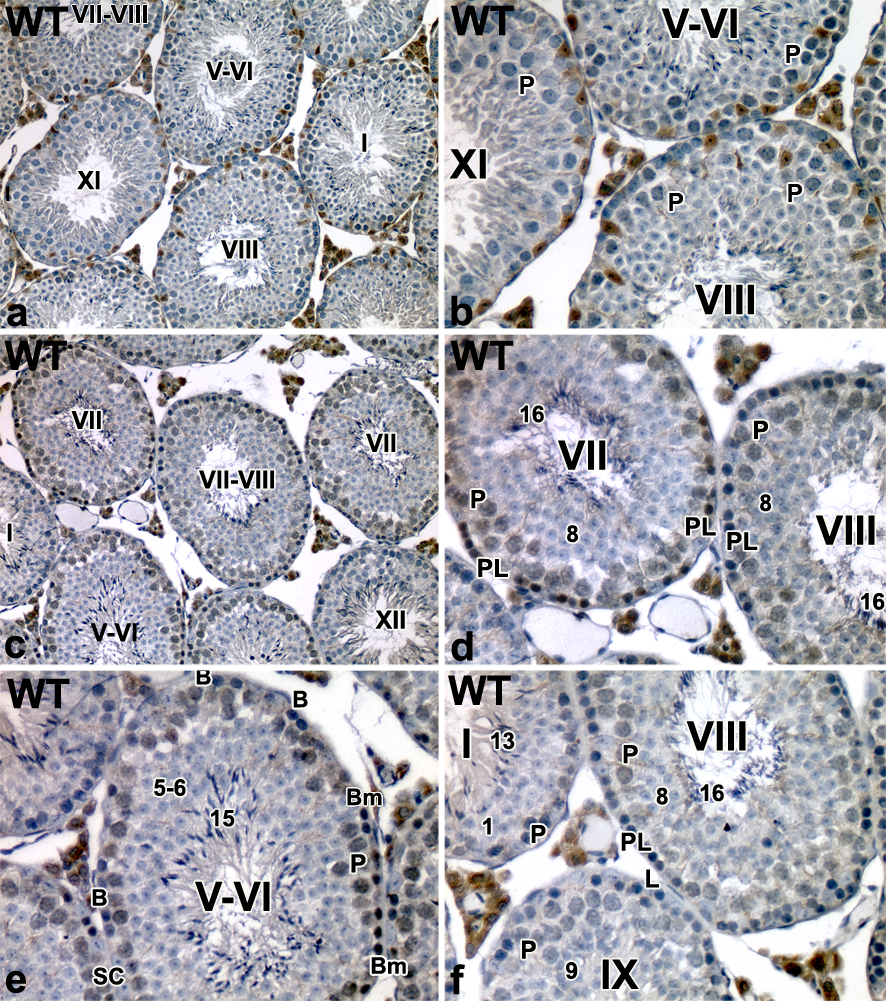

Supplement: Figure S1 — Cyclin E2 protein but not E1 was consistently found in pachytene spermatocytes at all stages. Histological sections of testes from adult wild type (WT) were immunostained with anti-cyclin E1 (a,b) and anti-cyclin E2 (c–f) antibodies. Magnification: a,c ×20; b, d–f ×40. B, B-type spermatogonia; Bm, dividing B-type spermatogonia; PL, preleptotene spermatocytes; L, leptotene spermatocytes; Z, zygotene spermatocytes; D, diplotene spermatocytes; P, pachytene spermatocytes. Arabic numerals indicate the step of spermatid differentiation; Roman numerals indicate the stage of the tubules. (TIF) [file pgen.1004165.s001.tif]

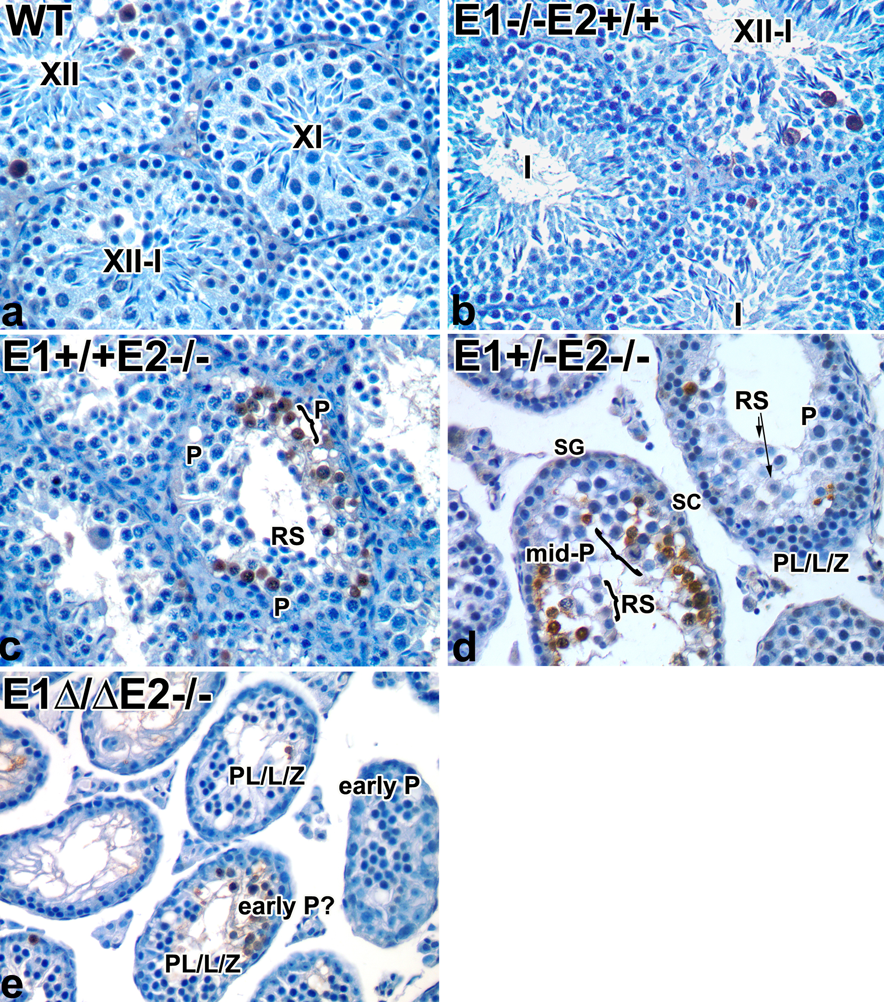

Supplement: Figure S2 — TUNEL-positive pachytene spermatocytes were found in the E-type cyclin deficient germline. Representative stages of seminiferous tubules containing TUNEL positive cells in wild type (WT, a), E1−/−E2+/+ (b), E1+/+E2−/− (c), E1+/−E2−/− (d) and E1Δ/ΔE2−/− (e) testes. The most striking observation was the presence of TUNEL-positive pachytene spermatocytes in both E1+/+E2−/− and E1+/−E2−/− testes (c,d), regardless of the severity of the testicular abnormalities (as reflected from the loss of advanced spermatogenic cells). In addition, TUNEL-positive spermatids were not detected. Magnification: a–e ×40. PL/L/Z, preleptotene-leptotene-zygotene spermatocytes; P, pachytene spermatocytes, early P, early pachytene spermatocytes; mid-P, mid pachytene spermatocytes; RS, round spermatids. Arabic numerals indicate the step of spermatid differentiation; Roman numerals indicate the stage of the tubules. (TIF) [file pgen.1004165.s002.tif]

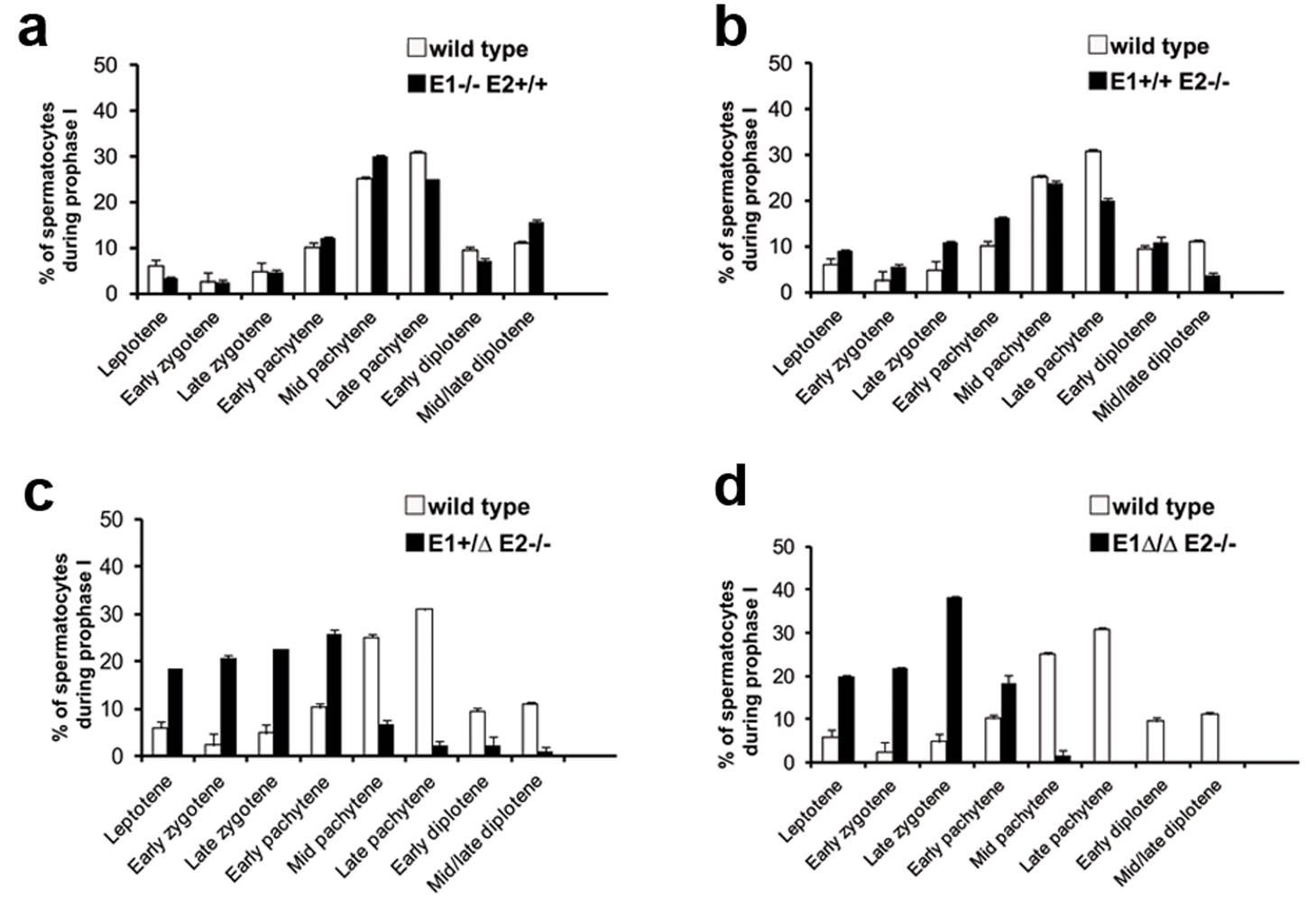

Supplement: Figure S3 — Percentage of spermatocytes present in each stage of prophase I. Wild type (WT) (white bars) and mutant (black bars) spermatocytes: E1−/−E2+/+ (a), E1+/+E2−/− (b), E1+/ΔE2−/− (c) and E1Δ/ΔE2−/− (d) spermatocytes. Each bar represents the mean number of spermatocytes obtained from one testis each from three mice per genotype. Per animal, a total of 400 (in E1+/ΔE2−/− and E1Δ/ΔE2−/− testis) and 500 spermatocytes (in all other genotypes) were counted. Error bars represent SEM. (TIF) [file pgen.1004165.s003.tif]

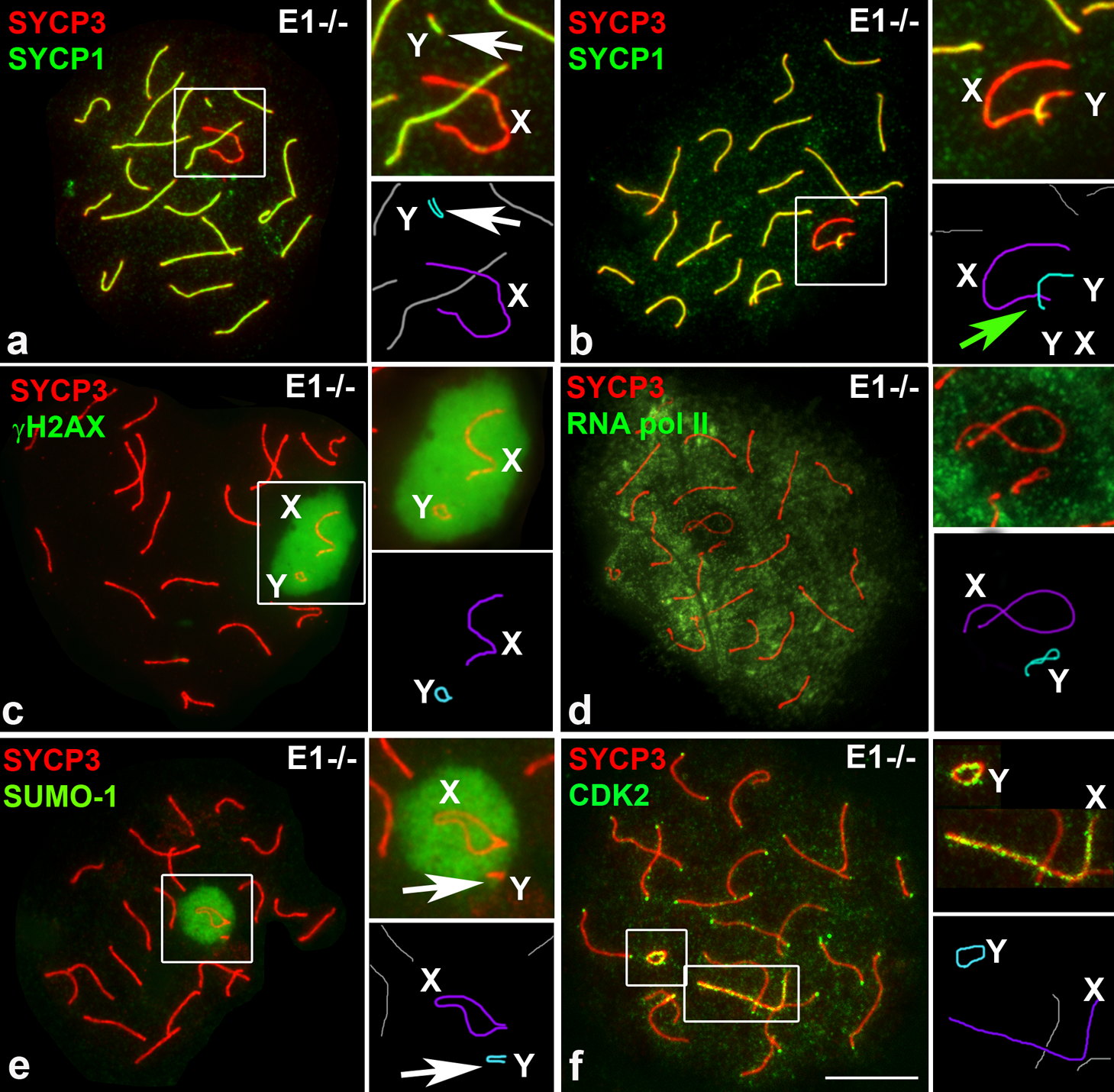

Supplement: Figure S4 — E1 depletion solely disrupts the synapsis of sex chromosomes. Chromosome spreads from E1−/−E2+/+ spermatocytes immunostained with SYCP3 (red) and SYCP1 (a–b); γH2AX (c), RNA pol2 (d), SUMO-1 (e) and CDK2 (f) (green). Insets represent the magnifications of the area selected in (a–f) (white squares) above their schematic representations. The X and Y chromosomes were frequently observed in total asynapsis (a,c,,d,f, insets) or in a peculiar synapsis that comprised only a small area in the pseudo-autosomal region (PAR) (b, green arrow). Y chromosome self-synapsis (insets in a,e, white arrows) or telomeres of the X or Y chromosome close together in a ring configuration (insets in c,e,f,) were observed. (TIF) [file pgen.1004165.s004.tif]

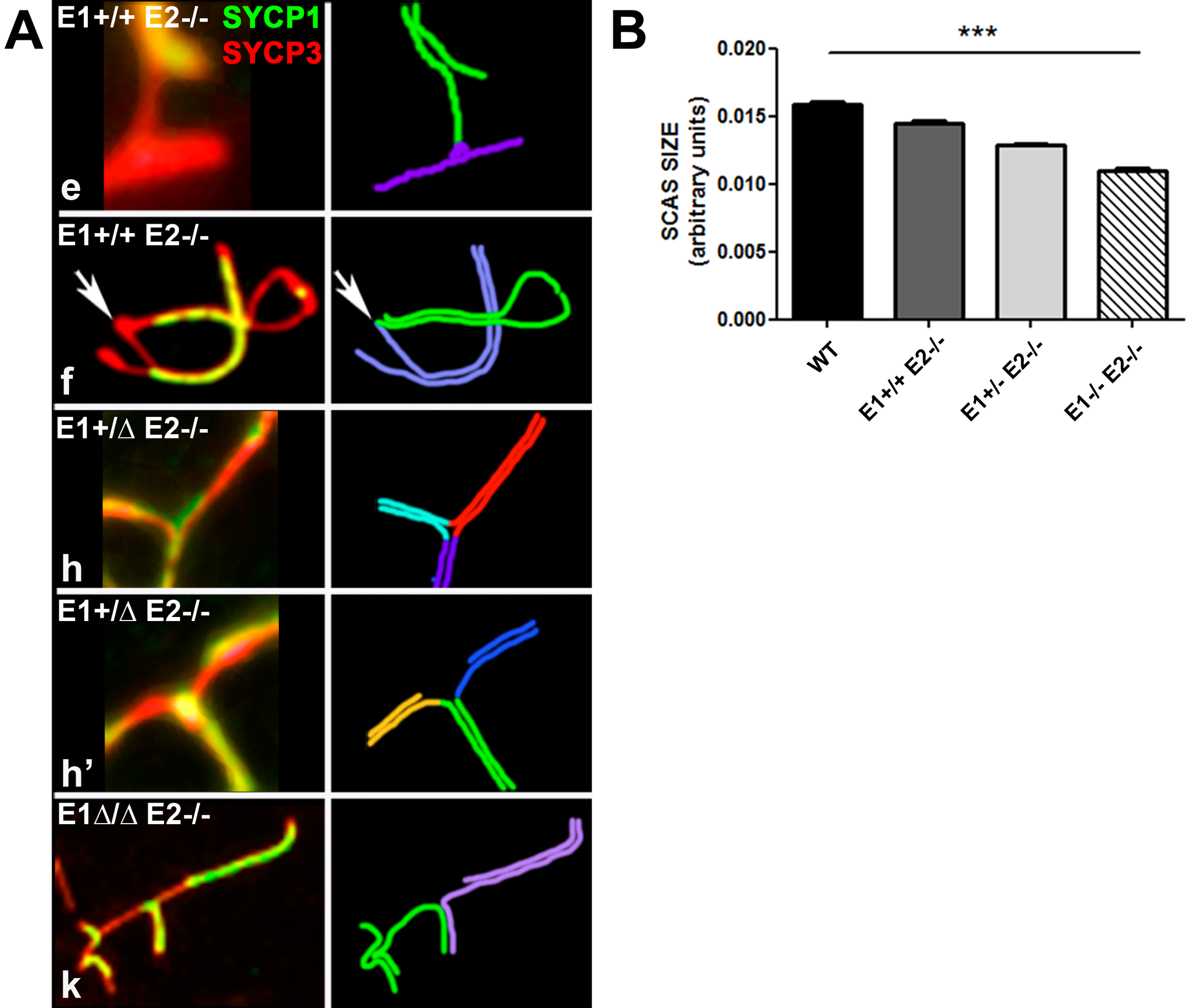

Supplement: Figure S5 — A) Schematic representations of white insets shown in Figure 4 and B) SCAS measurements. e) Insets and their respective schemes of Figure 4e showing the association of an autosomal end with the X chromosome. f) Inset and schematic of Figure 4f. Two heterologous autosomes are associated through their chromosome ends (white arrow). h–h′) Insets and schematics of Figure 4h. Three heterologous autosomes are associated through their chromosome end. k) Inset and schematic of Figure 4k. Two heterologous autosomes are partially synapsed. Each color represents a different chromosome in the insets. B) SCAS measurements. *** p≤0.001, n = 6 cells per genotype. (TIF) [file pgen.1004165.s005.tif]
