# Supplementary material for: Mammalian E-type Cyclins Control Chromosome Pairing, Telomere Stability and CDK2 Localization in Male Meiosis
Source: PLoS Genet. 2014 Feb 27;10(2):e1004165. doi: 10.1371/journal.pgen.1004165 (PMC3937215; doi:10.1371/journal.pgen.1004165)
Supplement: Table S1 — E2 depletion results in variable reduced sperm counts and numbers of fetuses/pups. E1+/+E2−/− (A), E1+/ΔE2−/− (B). n = 10 for both genotypes. (PDF) [file pgen.1004165.s006.pdf]

Table S1, related to Figure 3. Fertility studies

A. *E1+/+E2-/-* mice

Mouse (*E1+/+ E2-/-*)	Testis Weight (g)	Sperm Count ($x10^6$)	Mating 1 (fetuses+pups)	Mating 2 (fetuses+pups)	Mating 3 (fetuses+pups)
1			20	21	11
2			1	11	0
3			13	2	0
4			0	1	4
5	0.081	1.82	1	0	0
6	0.079	4.45	17	14	11
7	0.069	3.42	30	23	9
8	0.076	8.7	0	13	10
9	0.095	8.56	24	25	15

B. *E1+/-E2-/-* mice

Mouse (*E1+/- E2-/-*)	Testis Weight (g)	Sperm Count ($x10^6$)	Mating 1 (fetuses+pups)	Mating 2 (fetuses+pups)	Mating 3 (fetuses+pups)
1			0	0	0
2			0	0	0
3			0	0	0
4			0	0	0
5			0	0	0
6	0.029	0	0	0	0
7	0.056	0	0	0	0
8	0.03	0	0	0	0
9	0.03	0	0	0	0
10	0.027	0	0	0	0
